# Oil and Water Recovery from Palm Oil Mill Effluent: A Comparative Study of PVDF and α-Al_2_O_3_ Ultrafiltration Membranes

**DOI:** 10.3390/membranes15060176

**Published:** 2025-06-10

**Authors:** Saqr A. A. Al-Muraisy, Jiamin Wu, Mingliang Chen, Begüm Tanis, Sebastiaan G. J. Heijman, Shahrul bin Ismail, Jules B. van Lier, Ralph E. F. Lindeboom

**Affiliations:** 1 Sanitary Engineering Section, Department of Water Management, Faculty of Civil Engineering and Geosciences, Delft University of Technology, 2628 CN Delft, The Netherlandsm.chen-1@tudelft.nl (M.C.); m.b.tanis@tudelft.nl (B.T.); s.g.j.heijman@tudelft.nl (S.G.J.H.); j.b.vanlier@tudelft.nl (J.B.v.L.); r.e.f.lindeboom@tudelft.nl (R.E.F.L.); 2 Faculty of Ocean Engineering Technology, Universiti Malaysia Terengganu, Kuala Nerus 21030, Terengganu, Malaysia; shahrul.ismail@umt.edu.my

**Keywords:** palm oil mill effluent, polymeric membranes, ceramic membranes, oil recovery, water recovery

## Abstract

Recovering oil and water from palm oil mill effluent reduces environmental pollution and promotes sustainable practices. An effective method to achieve this is ultrafiltration (UF), which uses semi-permeable membranes to separate oil, solids, and other contaminants from wastewater under pressure. To assess the most effective recovery method, an experimental comparison was conducted between PVDF and α-Al_2_O_3_ UF membranes at constant permeate of 20–50 LMH for PVDF and 20–70 LMH for α-Al_2_O_3_ membranes. Both membranes achieved 99.8% chemical oxygen demand (COD) rejection, with oil concentration factor (F_o_) of 186.8% and 253.0%, and water recovery (R_w_) of 46.6% and 60.5%, respectively. The permeate water quality was superior to the Malaysian discharge standards, and the fat, oil, and grease (FOG) content was suitable for phase separation processes. The optimal permeate fluxes, with stable transmembrane pressures (TMP), were observed at 40 LMH (PVDF) and 60 LMH (α-Al_2_O_3_). Total resistance (R_t_) values were 1.30 × 10^12^ m^−1^ (PVDF) and 1.59 × 10^12^ m^−1^ (α-Al_2_O_3_). The ratio of irreversible to total resistances (R_ir_/R_t_) was 0.02 (PVDF) and 0.06 (α-Al_2_O_3_), indicating minimal irreversible fouling. Overall, the α-Al_2_O_3_ membrane demonstrated superior performance in oil and water recovery with more stable operation compared to the PVDF membrane. UF membrane technology emerges as an efficient technique for recovering oil and water compared to conventional methods.

## 1. Introduction

The global concern for the recovery of industrial effluents is increasing due to freshwater scarcity and environmental protection agreements [1,2,3,4]. Recently, more attention has been directed toward the discharge of oily wastewater, a major pollutant of the aquatic environment [4,5,6,7,8]. In this context, the removal of oil from palm oil industry waste streams is a major challenge, particularly in countries like Malaysia and Indonesia. Addressing this issue is crucial for developing more sustainable industrial practices [6,9]. Palm oil, identified as one of the rapidly growing industries due to its numerous product applications, continues to experience high demand [10,11]. Since 2016, global palm oil production has achieved continuous growth every year [10,12], simultaneously increasing its main brownish palm oil mill effluent (POME) [13,14].

POME is the primary waste stream generated during the processing of oil palm fresh fruit bunches (FFB) [15]. The latest reports show that global crude palm oil (CPO) production exceeded 79 million metric tons per year [16], which can be visualized by filling approximately 31,600 Olympic-sized swimming pools [17]. For every 1 ton of CPO produced, approximately 2.5 tons of POME are discharged [13]. POME typically contains 2.2 g/L–27.2 g/L of oil and grease [18], 11.5 g/L–79.0 g/L of total solids [18], and 894 g/L–986 g/L of water. The presence of residual oil and high organic matter in POME poses a serious environmental threat to receiving ecosystems [11]. The high organic matter content leads to elevated biological oxygen demand (BOD) and chemical oxygen demand (COD) levels in recipient surface waters. The discharge of water with high BOD and COD results in the rapid microbial consumption of dissolved oxygen, leading to the formation of anaerobic, or dead, zones in the surface water [11,19]. Further information on POME generation can be found in the literature [14,20,21].

The traditional ponding system, consisting of anaerobic or aerobic ponds, is insufficient in treating POME, as it requires a large open area and fails to meet discharge requirements or prevent environmental damage caused by oil adsorption and oxygen depletion [10,22]. For instance, the open ponding system can only remove approximately 95% of COD and BOD after a lengthy treatment period of 60 days or more [23], while the discharge requirements demand 99% and 99.5% removal of POME COD and BOD, respectively [14]. Moreover, the adsorption of oil residues on microbial surfaces leads to inhibition, biomass flotation, and washout of suspended solids [24]. Therefore, the presence of residual palm oil in rivers needs to be considered as a hazardous material for the environment that requires proper management [6,25,26]. At the same time, the oil content of POME holds potential value as it can serve as a raw material for products such as cosmetics, pharmaceuticals, and soap [27]. Recovered oil from POME can also be utilized as cooking oil or as concentrated feed for bioreactors, which reduces the reactor volume and surface area required for POME treatment.

The residual oil in POME can be categorized into four different particle size distributions (PSDs): free oil (>150 µm), dispersed oil mixture (20–150 µm), emulsified oil (5–20 µm), and soluble oil mixture (<5 µm) [11]. Free oil, which floats on the surface, can be easily removed using skimming or gravitational recovery technologies [11]. However, these conventional methods are insufficient for recovering oil from wastewater with low oil concentrations (i.e., <400 ppm) and smaller oil droplets (i.e., <20 μm) [6,28].

In addition to conventional open ponding systems [10,22], extensive research has been conducted on various technologies for treating POME, including biological wastewater treatment methods [10,22], Fenton-oxidation [29], coagulation and flocculation [30], and electrocoagulation [31,32]. Recent studies have also focused on resource recovery from POME, such as water reuse [14,33], residual oil recovery using an oil trap tank [34], and oil adsorption using polypropylene micro/nanofiber adsorbents [11]. However, the oil concentration in the effluent from these methods often exceeds the allowable discharge limits, making them unsuitable for disposal [6]. In addition, these techniques are not effective in separating low oil concentration wastewater (<400 ppm) and smaller oil droplets (<20 μm) [6,28]. Furthermore, these methods have limitations, including the requirement for a large operating surface area [6].

Membrane technology has proven to be a highly efficient method for recovering residual oil, achieving removal rates as high as 99.7%, significantly outperforming conventional approaches [4,6,7,14,35,36]. Ultrafiltration (UF) membranes, in particular, have been widely applied due to their ability to separate emulsified oil droplets and suspended solids effectively [6,37]. UF operates at low pressure, resulting in lower capital and operational costs compared to high-pressure membrane systems [6].

While several studies have demonstrated the effectiveness of UF in treating oily wastewater, challenges such as membrane fouling remain a concern [1,6,38]. The surface properties of the membrane play a crucial role in its antifouling performance [39]. In this line, Zhu et al. [40] showed that hydrophilic and oleophobic hollow fiber membranes significantly improved UF performance in separating oil and water from POME. Recent reviews by Aryanti et al. and Ho et al. provide an overview of membrane technologies applied in POME treatment, including progress in membrane materials, operating conditions, and fouling control through surface modifications and cleaning protocols [41,42].

There is growing evidence that UF membranes are particularly promising for POME treatment. For example, Ho et al. highlighted UF effectiveness in oil and water recovery when operated under optimized conditions [41]. Similarly, Samavati et al. investigated how variables such as pressure, temperature, and crossflow velocity influence membrane fouling and recovery efficiency during POME treatment, offering insights into process optimization [21]. Additionally, Aryanti et al. assessed advanced ceramic and polymeric UF membranes, identifying hybrid systems as a promising solution to the limitations of single-membrane processes [42].

While many studies have focused on reducing the fouling of UF membranes when treating oily wastewater, fewer studies have explored the oil and water recovery efficiency across different types of UF membranes. Polymeric membranes are commonly used in oily wastewater treatment due to their low manufacturing cost, ease of processing, and low energy requirements [1,7,43]. PVDF membranes offer lower upfront costs but may require more frequent replacement and cleaning, especially under harsh effluent conditions like those found in POME treatment, potentially increasing long-term operational expenditures [44]. However, they are more prone to fouling compared to ceramic membranes [1,7,43] and typically have limited thermal stability, with maximum operating temperatures generally below 60–70 °C, beyond which membrane deformation and degradation may occur [45]. Prolonged exposure to harsh cleaning agents, such as strong oxidizing solutions, can lead to degradation of PVDF membranes. For instance, a study demonstrated that PVDF membranes experienced a decline in performance after repeated cleaning cycles with sodium hypochlorite solutions, indicating potential chemical vulnerability under certain conditions [46].

Ceramic membranes, in contrast, exhibit greater fouling resistance, easier cleaning, lower maintenance, and higher mechanical strength [37,47,48,49,50]. They also offer excellent thermal and chemical resistance, allowing for stable operation at temperatures exceeding 90 °C without significant performance loss [37,47,48,49,50]. α-Al_2_O_3_ ceramic membranes exhibit superior chemical and thermal stability, making them more resilient in aggressive wastewater environments [37,47,48,49,50]. Recent advancements in ceramic membrane technology have highlighted their robustness and suitability for oil-water separation processes, including POME treatment [51,52]. These distinctions in chemical stability are critical when considering long-term operational efficiency and maintenance requirements. Moreover, ceramic membranes have a longer lifespan of around 20 years, whereas polymeric membranes typically last 7 to 10 years, resulting in lower life-cycle costs [7,43]. Therefore, ceramic membranes are used in various industrial applications, not only when polymeric membranes cannot perform properly, but also where high system integrity is needed [48]. However, the higher initial capital costs of ceramic membranes have limited their adoption in certain industrial sectors, such as food and beverage and oil and gas produced waters [43]. While ceramic membranes like α-Al_2_O_3_ typically have higher initial capital costs, ranging from 5 to 10 times that of PVDF, they are well known for their extended operational lifespan, superior chemical and thermal stability, and reduced cleaning frequency which are factors that can lead to lower overall lifecycle costs [53]. Therefore, the water industry is increasingly recognizing ceramic membranes as a viable and cost-competitive option in the long run, leading to growing industrial interest in their applications [43,48].

Most industrial UF systems operate at a constant permeate flux to maintain a consistent production rate of permeate water [54]. This approach allows for the adjustment and control of permeate flux to maximize water production, oil concentration, and recovery while minimizing fouling [54]. Moreover, UF membranes are more stable and require less frequent cleaning when operated in the constant permeate flux mode compared to constant transmembrane pressure (TMP) mode [54,55]. Therefore, this study adopts the constant permeate flux mode of filtration.

While some research has been conducted on ceramic UF membranes for oily wastewater treatment [6,51,52,56], there remains a critical gap in quantitative data on the recovery of oil and water and the operational performance of POME UF using ceramic membranes compared to their polymeric counterparts. In this regard, the objectives of the present study are as follows: (i) Analyze the performances of PVDF and α-Al_2_O_3_ membranes in terms of permeate flux, rejection capacity, fouling tendency, and the efficiency of membrane cleaning to recover hydraulic permeability. (ii) Determine the optimal operating conditions for both membranes within the specified range of variables, and (iii) Evaluate the quality of the recovered oil and permeate water for potential reuse applications, comparing them to relevant standards.

Ceramic membranes are typically capable of operating at higher temperatures [47], presenting an opportunity for thermal energy recovery, considering that POME is typically discharged at temperatures between 80 and 90 °C [57]. With the collective efforts towards enhancing the sustainable practices of palm oil production and maximizing the utilization of resources in waste streams, this study serves as a foundation for future POME treatment and resource recovery.

## 2. Materials and Methods

### 2.1. POME Emulsion Preparation

In the laboratory, POME emulsion was synthesized to replicate the main characteristics, including the particle size distribution (PSD) of oil droplets and the oil content. The PSD of oil droplets in oily wastewater has been reported to have an average range of 0.8 µm–1.4 µm [58], while the oil content in POME was measured on-site and found to be in the range of 2.2 g/L–27.2 g/L [18]. To achieve similar properties, unrefined palm oil (UPO) (KTC Edibles, Wednesbury, UK) was mixed with demineralized water at an initial concentration of 6 g UPO/L. The mixture was heated at 55 °C and simultaneously shaken at 150 RPM using an incubator shaker (New Brunswick Innova 43, Eppendorf, Hamburg, Germany) for 24 h. To enhance the emulsion process and minimize the hydrophobic characteristics of the unrefined palm oil, the mixture was further sonicated using an energy-intensive sonifier (Branson Digital Sonifier 450, Branson Ultrasonics, Brookfield, CT, USA) for 30 min at 40% intensity. The mixture was left to cool down to room temperature, then sieved using a 0.103 mm sieve (INTERLAB-BV, Putte, The Netherlands) to remove large oil aggregates. The oil content was measured via COD kits (Hach Lange, Loveland, CO, USA), and the PSD of oil droplets was analyzed with a particle size analyzer (Bluewave, Microtrac, York, PA, USA). POME emulsion was placed in a 10 L feed container prior to the experiments. It was used fresh for the UF experiments, after being cooled to room temperature for 2 h–4 h, to prevent any degradation or alteration of its physicochemical properties.

In this study, the prepared POME emulsion represents a solids-free, pretreated version of POME to prevent rapid membrane fouling caused by the high solids content present in raw POME. Pretreatment is essential to remove solids, protect the longevity and integrity of the membrane system [14,59,60,61], and focus on the recovery of oil and water during UF experiments.

### 2.2. Experimental Setup

Two types of tubular UF membranes were used in the present study: polymeric PVDF (Pentair, Golden Valley, MN, USA) and ceramic α-Al_2_O_3_ (Inopor, Veilsdorf, Germany). Table 1 illustrates the properties of the membranes. PVDF and α-Al_2_O_3_ membranes were installed in thermoplastic PVC-C and stainless-steel housings, respectively. Both membranes were operated in an inside-out configuration.

**Figure 1:**
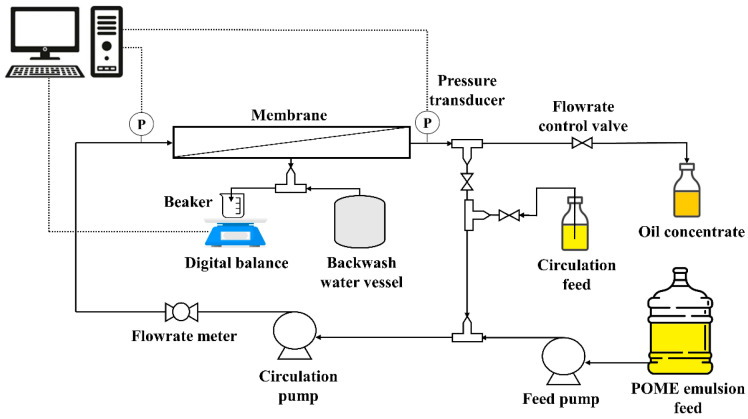
Schematic diagram of the ultrafiltration (UF) setup of POME emulsion using PVDF and α-Al_2_O_3_ membranes at a crossflow velocity (CFV) of 0.8 m/s.

A laboratory-scale constant permeate flux filtration system, compatible with both PVDF and α-Al_2_O_3_ membrane modules, was employed for the experiments (Figure 1). The system comprised two distinct pumps serving separate functions. The feed pump, a compact positive displacement diaphragm dosing pump (DDA12-10, Grundfos, Bjerringbro, Denmark), supplied the POME emulsion from the main feed container into the filtration circuit and controlled the permeate flux. Permeate flux was maintained at a constant setpoint via an integrated flow controller with a closed-loop feedback mechanism that continuously adjusted the pump speed based on real-time permeate flow rate measurements, thereby preventing the need for manual adjustments during operation.

Crossflow velocity (CFV) was regulated by a rotary gear circulation pump (VerderGear R, Verder Liquids, Utrecht, The Netherlands), which recirculated the feed solution from a separate circulation feed vessel through the membrane module. The flow rate within the circulation loop was continuously monitored using a flowmeter (YF-S402, Zhongjiang Energy-Efficient Electronics, Foshan, Guangdong, China) to ensure stable hydrodynamic conditions. The oil concentrate stream exiting the membrane module was collected separately after the end of each experiment and was not returned to the feed vessel, thereby maintaining a constant volume within the circulation loop.

To mitigate membrane fouling and maintain performance, a backwashing step was performed after each filtration cycle. Backwashing was carried out for 30 s at a fixed pressure of 3 bar using demineralized water supplied from a dedicated vessel pressurized by compressed air.

Two pressure transducers (GS4200-USB, ESI Technology Ltd., Wales, UK) were connected to the two sides of the membrane module to monitor the filtration pressure continuously with a time interval of 30 s. The permeate water was weighed using an online digital balance (EWJ 600, Kern & Sohn GmbH, Balingen, Germany). Pressure transducers and the flow rate sensor were logged during the experiments, and data were monitored and recorded in real-time using DASYLab software (version 13.0). Since the permeate pressure was under atmospheric pressure, the values displayed by the transducers were taken as the transmembrane pressure (TMP), as described in the literature [65]. Further operational details of the setup can be found in [65].

### 2.3. Experimental Design

#### 2.3.1. Constant Permeate Flux Crossflow Experiments

Filtration experiments were conducted at room temperature (~22 °C) with a constant permeate flux for each test. The steady-state TMP was typically reached within 1.5–2 min after the start of the filtration cycle. For the PVDF membrane, the permeate flux was incrementally increased starting from 20 LMH to 50 LMH. Due to its larger pore size of 70 nm, it was expected that the α-Al_2_O_3_ membrane would exhibit higher hydraulic permeability in comparison to the PVDF membrane. Consequently, the α-Al_2_O_3_ membrane was also expected to achieve a higher optimal permeate flux. Therefore, the α-Al_2_O_3_ membrane was tested at permeate fluxes ranging from 20 LMH to 70 LMH. The maximum flux for both membranes was determined based on the stability of TMP and the maximum pressure endured by the feed pump. Table 2 provides a summary of the operational parameters for each membrane.

To achieve and maintain constant flux operation, the system utilized a feed pump with an integrated flow controller, which actively measured and regulated the volumetric flow rate to meet the set flux targets. The flow rate was calibrated according to the membrane surface area to achieve the desired LMH. During filtration, any change in membrane resistance due to fouling was compensated for by the pump adjusting its power output to sustain the target permeate flow [65,66].

To determine the optimal permeate flux within the test range, the following criteria were considered: (1) maintaining the maximum TMP between 1 bar and 1.5 bar, (2) achieving the highest stable permeate flux that yields the maximum oil concentration factor (F_o_) and water recovery (R_w_), (3) minimizing TMP variation and irreversible fouling to reduce the frequency of chemical cleaning and extend membrane lifespan, and (4) ensuring a minimum irreversible resistance over filtration time, expressed as the ratio of irreversible to total resistances (R_ir_/R_t_) of ≤0.1.

Each UF experiment consisted of three filtration cycles and two hydraulic permeability tests using demineralized water, with the first test conducted before the first filtration cycle and the second test performed after the third filtration cycle. Further details on the filtration phases and hydraulic permeability tests are available in the Supplementary Materials (Section S1). Permeate water samples of the optimum UF condition were characterized. These characteristics of permeate water collected during the filtration cycles were measured in triplicates, and standard deviation was reported (Table 4).

**Table 2:** Operational parameters of PVDF and α-Al_2_O_3_ membranes.

Parameter/Membrane	CFV (m/s)	Flux 1 (LMH)	Flux 2 (LMH)	Flux 3 (LMH)	Flux 4 (LMH)
PVDF	0.8	20	40	50	-
α-Al_2_O_3_	0.8	20	40	60	70

#### 2.3.2. Membrane Conditioning and Cleaning

Prior to the experiments, both PVDF and α-Al_2_O_3_ membranes were soaked in a 25% *v*/*v* ethanol solution (Sigma-Aldrich, St. Louis, MO, USA) for two hours to remove any residuals in the membrane wall and pores [67]. They were then washed with demineralized water [67].

After each UF experiment, the membranes underwent both physical and chemical cleaning. Physical cleaning involved flushing the filtration loop with demineralized water at the same crossflow velocity (CFV) used during the UF experiment until the water discharged from the outlet was clean.

Chemical cleaning was performed outside the system, using the membrane module after it was removed from the filtration setup. The PVDF membrane module was soaked in a freshly prepared 0.1 M NaOH solution (Sigma-Aldrich, St. Louis, MO, USA) for 12–15 h at room temperature [67]. The α-Al_2_O_3_ membrane module was similarly soaked in a 0.75 M NaOH solution (Sigma-Aldrich, Missouri, USA), which was maintained at 40 °C throughout the 12–15 h soaking period [68]. No transmembrane pressure, crossflow, or mixing was applied during chemical cleaning; the process relied solely on static soaking. The volume of the used cleaning solution matched the internal volume of the membrane module to ensure full submersion of the membrane. To ensure consistent cleaning conditions and reproducibility of the results, fresh NaOH solution was used for each cleaning cycle and was not reused.

Following chemical cleaning, the membrane modules were rinsed thoroughly with demineralized water before being reinstalled in the system. The effectiveness of both physical and chemical cleaning steps was evaluated by monitoring the recovery of the membranes hydraulic permeability.

#### 2.3.3. Membrane Performance Evaluation Methods

##### Rejection Capacity (R_j_)

The treatment efficiency of the membranes can be directly evaluated using the rejection rate, R_j_, as shown in Equation (1).

(1) $$R_{j}\;\left(\%\right)=\left(1-\frac{C_{p}}{C_{f}}\right)\times 100$$

where C_p_, C_f_ are the concentrations of parameters of interest in permeate water and feed, respectively. In this study, the rejection capacity is calculated for chemical oxygen demand (COD), total solids (TS), turbidity, and electrical conductivity (EC) of feed and permeate water.

##### Oil Concentration Factor (F_o_)

The oil concentration factor (F_o_) measures the oil concentration and indirectly indicates the potential of oil recovery from the POME concentrate. Analyzing the quality of the recovered oil through F_o_ helps to determine any necessary applications or post-treatments. F_o_ is calculated by dividing the COD ratio of the oil in the POME concentrate by that in the POME emulsion feed (g concentrate COD/g POME emulsion COD feed) (Equation (2)). The COD value of the POME concentrate (COD_conc._) was determined by the equation of COD balance (Equation (3)). The equation represents the total COD involved before and after the filtration experiment with COD_conc._ calculated based on the assumption of a homogeneous POME concentrate.

(2) $$F_{o}=\frac{{\mathrm{COD}}_{\mathrm{conc}.}}{{\mathrm{COD}}_{f}}\times 100\%$$

(3) $${\mathrm{COD}}_{f}\cdot \left({\dot{V}}_{f}\times t_{\mathrm{cycle}}+V_{\mathrm{loop}}\right)={\;\mathrm{COD}}_{\mathrm{conc}.}\times V_{\mathrm{loop}}+{\;\mathrm{COD}}_{P}\times {\dot{V}}_{p}\times t_{\mathrm{cycle}}$$

where COD_f_ is the COD of POME emulsion feed (g POME COD/L), COD_conc._ is the COD of the POME concentrate discharged from the filtration system (g concentrate COD/L), and COD_p_ is the COD of the permeate water (g permeate water COD/L). ${\dot{V}}_{f}$ is the volumetric flowrate of POME emulsion fed into the filtration system (L/min). ${\dot{V}}_{p}$ is the volumetric flowrate of the permeate water (L/min). V_loop_ is the total volume (L) of the filtration system, i.e., the total internal volume of the tubes, connections, and membrane. V_loop_ using PVDF and Al_2_O_3_ membranes was calculated at 0.12 L and 0.13 L, respectively. t_cycle_ is the time for each filtration cycle (15 min).

##### Water Recovery (R_w_)

R_w_ represents the ratio of permeate water volume to the total POME emulsion feed volume (L permeate water/L POME emulsion feed) (Equation (4)).

(4) $$R_{w}\;\%=\frac{{\dot{V}}_{p}\times t_{\mathrm{cycle}}}{{\dot{V}}_{f}\times t_{\mathrm{cycle}}+{\;V}_{\mathrm{loop}}}\times 100\%$$

##### Normalized Transmembrane Pressure (TMP_n_)

TMP_n_ was calculated using Equation (5). This method is adopted from the literature [65,69].

(5) $${\mathrm{TMP}}_{n}=\frac{\mathrm{TMP}}{{\mathrm{TMP}}_{0}}$$

where TMP is the POME emulsion UF transmembrane pressure (Pa) at a specified filtration time, and TMP_0_ is the membrane initial transmembrane pressure (Pa) using demineralized water.

#### 2.3.4. Membrane Fouling Evaluation

Resistance-in-series model [67,70], using Darcy’s law (Equation (6)), was adopted to evaluate membrane fouling via membrane resistances. Further details on this method and the equations used are available in the Supplementary Materials (Section S3).

(6) $$R_{t}=\frac{\mathrm{TMP}}{\mu J}={\;R}_{m}+R_{r}+R_{\mathrm{ir}}$$

where R_t_ (m^−1^) is the total resistance comprising intrinsic membrane resistance (R_m_, m^−1^), hydraulic reversible resistance (R_r_, m^−1^), and irreversible resistance (R_ir_, m^−1^). TMP represents the transmembrane pressure of POME emulsion filtration, and J is the permeate flux (m/s). The dynamic viscosity of the permeate water (µ) is assumed to be the same as that of pure water, 9.544 × 10^−4^ Pa·s, at the filtration temperature of 22 °C [54]. R_ir_/R_t_ ratio was used in this study to analyze and compare the degree of irreversible fouling that occurred under each UF condition.

##### Hydraulic Permeability

The hydraulic permeability (L_h_) was calculated using Darcy’s Law (Equation (7)).

(7) $$L_{h}=\frac{J}{{\mathrm{TMP}}_{0}}$$

where J is the permeate flux of demineralized water (LMH), and TMP_o_ is the transmembrane pressure (Pa) using demineralized water.

### 2.4. Analytical Methods

POME emulsion feed and permeate water samples were characterized in triplicates. Total and soluble chemical oxygen demand (COD_t_, and COD_s_) were determined using Hach-Lange kits (Hach Lange, USA). Fat, oil, and grease (FOG) content was directly calculated based on the measured COD of POME emulsion using the theoretical ratio of 2.71 g COD/g FOG. More details on the ratio calculation can be found in the Supplementary Materials (Section S2).

pH was measured using a multi-meter (Multi 3430, WTW inoLab_IDS, Xylem Analytics, Weilheim, Germany), whereas electrical conductivity (EC) was measured using a digital meter (Multi 9420, WTW inoLab_IDS, Xylem Analytics, Germany). Turbidity was analyzed using a digital turbidimeter (2100 N, Hach Lange, USA).

The particle size distribution of the oil droplets was determined using a particle size analyzer (S3500 Bluewave, Microtrac MRB, Haan/Duesseldorf, Germany). Total nitrogen and ammoniacal nitrogen were characterized using Hach-Lange kits (Hach Lange, USA). The measurements of total solids (TS) and total suspended solids (TSS) were performed according to the standard methods [71], whereas total dissolved solids (TDS) were calculated from the difference between TS and TSS. The analysis of these parameters is essential to assess the efficiency of the UF experiments using PVDF and α-Al_2_O_3_ membranes. Additionally, these analyses allowed for a comparison of the permeate water quality with industrial water discharge standards [72,73].

## 3. Results and Discussion

### 3.1. POME Emulsion Characteristics

The physicochemical characteristics of POME emulsion compared to raw POME are presented in Table 3. The POME emulsion has an acidic pH of 5.4, which is consistent with the reported literature on raw POME pH ranging from 3.4 to 5.5 [18]. The high amount of organic matter in the POME emulsion can be observed by COD_t_ of 15.0 ± 0.2 gO_2_/L, which is solely attributed to unrefined palm oil. Similarly, the concentration of fat, oil, and grease (FOG) in POME emulsion was found to be 5.5 ± 0.1 g/L, falling within the range of raw POME FOG values of 2.2 g/L–27.2 g/L [18]. COD of raw POME typically exhibits a higher COD range of 15 g/L–100 g/L [18], compared to the POME emulsion prepared in this study. Raw POME also contains other constituents such as protein (0.5 g/L–1.6 g/L), cellulose (4.0 g/L–14.3 g/L), lignin (9.0 g/L–15.2 g/L), extractives (4.4 g/L–14.5 g/L), and ash (1.2 g/L–5.9 g/L) [18].

The turbidity of POME emulsion was measured at 580.7 ± 9.0 NTU, which is lower than the turbidity of raw POME, in the range of (65.6–69.4) × 10^3^ NTU [18]. Other physicochemical characteristics of POME emulsion were also quantitatively lower than those of raw POME. For instance, EC of POME emulsion was measured at 18.3 ± 0.1 µS/cm, which is less than 137 µS/cm of raw POME [74]. Similarly, total nitrogen and ammoniacal nitrogen of POME emulsion of (5.9 ± 0.1) × 10^−3^ g/L, and 0.2 × 10^−3^ g/L, respectively, were lower than the respective values in raw POME, in the range of 0.2–1.7 and (17–254) × 10^−3^ g/L, respectively [18].

The presence of TDS at 1.8 ± 0.1 g/L in the POME emulsion, along with EC, was associated with monovalent and divalent ions, which could be from the dissociation of FOG [70]. Moreover, the TSS of 1.9 ± 0.1 g/L in the POME emulsion could be attributed to the colloids and suspended organic matter [70]. Furthermore, approximately two-thirds of the droplet size of the POME emulsion was 1.54 µm, which is consistent with the average particle size of oily wastewater [58]. The synthesis of POME emulsion with such a small particle size distribution of oil droplets is crucial for investigating the efficiency of oil and water recovery using PVDF and α-Al_2_O_3_ membranes, especially considering the challenges associated with recovering emulsified and soluble oil using conventional methods [6].

**Table 3:** Physicochemical characteristics of POME emulsion.

Parameters	POME Emulsion	Raw POME [18,74]
pH	5.4	3.4–5.5
Electrical Conductivity (µS/cm)	18.3 ± 0.1	137
Turbidity (NTU)	580.7 ± 9.0	(65.6–69.4) × 10^3^
COD_t_ (g/L)	15.0 ± 0.2	15–100
COD_s_ (g/L)	2.1	-
TS (g/L)	3.7 ± 0.1	11.5–79.0
TDS ^a^ (g/L)	1.8 ± 0.1	20.6–41.1
TSS (g/L)	1.9 ± 0.1	5–71.3
FOG ^b^ (g/L)	5.5 ± 0.1	2.2–27.2
Total nitrogen (g TN/L)	(5.9 ± 0.1) × 10^−3^	0.2–1.7
Ammoniacal nitrogen (g NH_4_-N/L)	0.2 × 10^−3^	(17–254) × 10^−3^
Particle size distribution (PSD) (µm, % *v*/*v*)	1.54 (61.5%), 0.04 (38.5%)	-

^a^ Value of POME emulsion was calculated from the difference between TS and TSS. ^b^ Value of POME emulsion was calculated using the ratio of 2.71 g COD_t_/g FOG.

A comparison between PVDF and α-Al_2_O_3_ membranes was made based on various performance variables, including the rejection capacity (R_j_), oil concentration factor (F_o_), water recovery (R_w_), the effect of permeate flux on normalized TMP (TMP_n_) and fouling resistances (R), and the efficiency of membranes cleaning. The key results of this comparison are summarized in Table 5.

### 3.2. Comparison on the Quality of Oil and Water Recovery

#### 3.2.1. Rejection Capacity (Rj)

**Figure 2:**
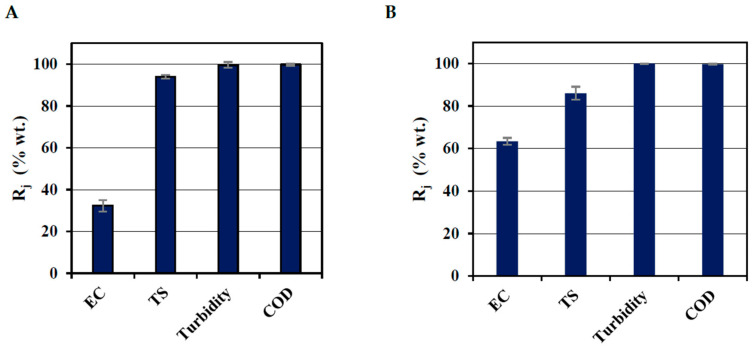
Steady-state rejection capacity (R_j_, % wt.) of EC, TS, turbidity, and COD at the optimum UF conditions, (**A**) PVDF membrane at 40 LMH, and (**B**) α-Al_2_O_3_ membrane at 60 LMH.

Rejection capacity for COD, turbidity, TS, and EC was analyzed at the optimum UF conditions for each membrane, as shown in Figure 2. Both membranes achieved high rejection rates for the selected parameters. Specifically, COD rejection of 99.8% was achieved with both membranes. Similarly, PVDF and α-Al_2_O_3_ membranes removed 99.6% and 99.9% of turbidity, respectively. The COD rejection obtained in this study was higher than that reported in the literature [70,75,76,77]. Hernández et al. [70] reported COD rejection of 80–85% and turbidity rejection of 90–95% using nanofiltration (NF) membranes. Moreover, Huang et al. [78] achieved a turbidity rejection of 98.3% for raw surface water using UF membranes. The high COD and turbidity rejections observed in this study suggest that suspended particles and colloids larger than the membrane’s pore sizes may be the primary foulants, which aligns with the findings of Huang et al. [78].

Furthermore, PVDF and α-Al_2_O_3_ membranes removed 94.0% and 86.1% of TS, respectively. It can be inferred that some oil droplets were smaller (0.04 µm) than the α-Al_2_O_3_ membrane pore size (0.07 µm), allowing them to pass through the membrane pores. Additionally, oil droplets may have been distorted during filtration, breaking into smaller particles that passed through the membrane pores [69,79,80]. However, rejections higher than 80% are considered adequate [70]. Furthermore, it was anticipated that the formation of an oil layer on the membrane surface would provide additional resistance to the passage of solutes [1]. This phenomenon was observed by the increasing values of rejection capacities from the first to the third filtration cycles using both membranes.

Rejection of EC was achieved at 32.2% and 63.4% using PVDF and α-Al_2_O_3_ membranes, respectively. The observed low EC rejection might be attributable to the fact that UF membranes have the ability to retain only insoluble suspended solids, colloids, and soluble macromolecular substances that are larger than the membrane pore size [69,70], but not mono acids such as volatile fatty acids (VFAs) [69]. Therefore, POME emulsion was analyzed for VFAs and found that it contained 2.2 g/L–4.6 g/L acetic acid, 0.4 g/L–1.2 g/L propionic acid, and 0.5 g/L–1.2 g/L butyric acid. VFAs in POME emulsion could be formed due to the breakdown of lipids during the sonication of unrefined palm oil (UPO). Nonetheless, EC rejection achieved in this study was higher than some reported values of 20% using NF membranes [70]. This enhanced rejection may be explained by the formation of an oil layer on the membrane surface driven by electrostatic and drag forces, creating steric hindrance that improves retention beyond what the membrane’s nominal pore size would suggest. Further details are available in the Supplementary Material (Section S4).

#### 3.2.2. Oil Concentration Factor (F_o_) and Water Recovery (R_w_)

**Figure 3:**
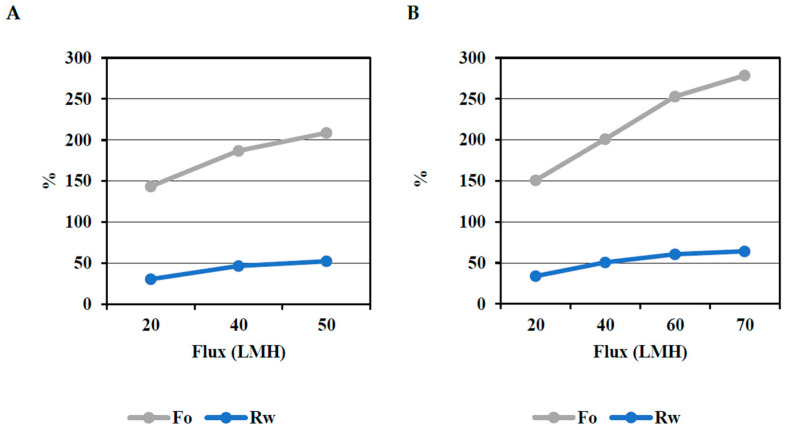
Oil concentration factor (F_o_), and water recovery (R_w_) at CFV = 0.8 m/s. (**A**) PVDF membrane, and (**B**) α-Al_2_O_3_ membrane.

F_o_ and R_w_ were analyzed at the operating conditions chosen for PVDF and α-Al_2_O_3_ membranes. As illustrated in Figure 3, F_o_ is proportionally related to R_w_ for both membranes.

Figure 3 also illustrates that F_o_ and R_w_ are both influenced by the rejection of oil droplets and were found to be proportional to the permeate flux. PVDF membrane achieved F_o_ ranging from 143.4% at 20 LMH to 208.6% at 50 LMH. Consequently, R_w_ ranged from 30.4% at 20 LMH to 52.2% at 50 LMH. At the optimum condition of 40 LMH, the PVDF membrane achieved R_w_ of 46.6%, resulting in F_o_ of 186.8%.

Comparatively, α-Al_2_O_3_ membrane achieved F_o_ ranging from 150.7% at 20 LMH to 278.6% at 70 LMH, which resulted in R_w_ ranging from 33.8% at 20 LMH to 64.1% at 70 LMH. It can be observed that the α-Al_2_O_3_ membrane obtained higher F_o_ and R_w_ at similar UF conditions compared to the PVDF membrane. For instance, at 40 LMH, F_o_ and R_w_ of 186.8% and 46.6%, respectively, were achieved for the PVDF membrane and 200.9% and 50.5% for the α-Al_2_O_3_ membrane. At the optimum condition of 60 LMH, the α-Al_2_O_3_ membrane achieved R_w_ of 60.5%, resulting in an F_o_ of 253.0%. According to Ren, et al. [69], F_o_ of 500% could be achieved using the α-Al_2_O_3_ membrane at a higher permeate flux, but this is on account of process stability, i.e., constant TMP_n_ and permeate flux over filtration time [81]. Furthermore, F_o_ is dependent on the feed and the membrane characteristics.

Under the optimum UF conditions, a COD_conc._ of 17.1 gO_2_/L and 20.4 gO_2_/L was calculated, corresponding to a FOG content of 6.3 g/L and 7.5 g/L for PVDF and α-Al_2_O_3_ membranes, respectively. This observation indicates that the POME concentrate could be suitable for traditional phase separation processes (gravity separation, centrifugation, hydrocyclones, and gas flotation, among others) since the oil concentration exceeds 400 ppm [6,28,37]. Another potential application for the recovered POME concentrate is biodegradation via anaerobic digestion to produce methane-rich biogas. Reducing the water content in the POME emulsion by 46.6% and 60.5% for PVDF and α-Al_2_O_3_ membranes, respectively, can decrease the bioreactor volume and surface area, or the membrane filtration area in the case of membrane bioreactor application, and subsequently reduce capital and operational costs.

#### 3.2.3. Permeate Water Characteristics

Permeate water quality characteristics at the optimum conditions of both membranes were analyzed and reported in Table 4. Moreover, industrial water discharge requirements were aimed at comparing the recovered water with the global standardized requirements for industrial water discharge. Overall, the treatment of POME emulsion exhibited high COD rejection capacities ranging from 94.0% to 99.8% using the PVDF membrane and 86.1% to 99.8% using the α-Al_2_O_3_ membrane, as illustrated in Figure 2.

The quality of permeate water using PVDF and α-Al_2_O_3_ membranes showed low values of turbidity and COD of 0.5–2.5 NTU and 33.1 mg/L–35.7 mg/L, respectively, demonstrating the retention of colloidal particles. Similar trends were observed for TS, TSS, FOG, ammoniacal nitrogen, and total nitrogen, where high retention was achieved using both membranes. A similar observation was reported in the literature for UF-RO treatment of food industry wastewater [70]. Although EC rejection was the lowest parameter, ranging from 32.2% to 63.4%, the permeate water still exhibited low EC values. The remaining EC in the permeate water could be attributed to the presence of volatile fatty acids and/or dissociated acids that the membranes’ MWCOs are not able to retain [70]. This was further supported by the TDS concentration in the permeate water, which reached 186.0 ± 44.2 mg/L and 488.5 ± 42.4 mg/L using PVDF and α-Al_2_O_3_ membranes, respectively. TDS could be associated with monovalent ions originating from the dissociation of FOG [70]. The slightly acidic permeate water, with a pH of 5.4 when using PVDF membranes and 6.0 with α-Al_2_O_3_ membranes, also contributed modestly to the observed EC value.

It is worth noting that all quality parameters are below the discharge limits of industrial water and specifically POME discharge standards, whereas pH was within the accepted range, as shown in Table 4. Therefore, the produced permeate water can be considered to have high-quality characteristics. The permeate water could be reused as boiler feed water in the palm oil mill, as service water within the mill, or potentially as a source for drinking water production [73]. It can also be discharged into the river system [73]. These findings indicate that the UF process to treat POME emulsion is a viable option for recovering clean water and oil.

**Table 4:** Comparison of permeate water characteristics of POME emulsion UF using PVDF and α-Al_2_O_3_ membranes.

Parameters	Permeate Water Characteristics		Industrial Water Discharge Requirements	POME Discharge Standards [14,82]
	PVDF Membrane	α-Al_2_O_3_ Membrane		
pH	5.4 ± 0.20	6.0 ± 0.1	6.5–8.5 [70,83]	5–9
EC (µS/cm)	12.4 ± 0.1	6.7 ± 0.1	260 [70]	-
Turbidity (NTU)	2.5 ± 0.04	0.5 ± 0.1	<5 [70]	-
COD (mg/L)	35.7 ± 1.5	33.1 ± 1.8	<50 [70]	<1000
TS (mg/L)	219.3 ± 50.0	509.0 ± 43	<1000 [70]	<1500
TSS (mg/L)	33.3 ± 5.8	20.3 ± 0.6	<400 [83]	<400
TDS ^a^ (mg/L)	186.0 ± 44.2	488.5 ± 42.4	-	-
FOG ^b^ (mg/L)	13.2 ± 0.6	12.2 ± 0.7	<50 [83]	<50
Ammoniacal nitrogen (mg NH_4_-N/L)	<0.001	<0.001	-	<100
Total nitrogen (mg TN/L)	<0.001	<0.001	<150 [83]	-

^a^ Value calculated from the difference between TS and TSS. ^b^ Value calculated using the ratio of 2.71 g COD/g FOG.

### 3.3. Comparison of the Rate of Oil and Water Recovery

#### 3.3.1. Effect of Permeate Flux on Normalized Transmembrane Pressure (TMP_n_)

UF experiments at a constant permeate flux of POME emulsion were conducted using PVDF and α-Al_2_O_3_ membranes. PVDF membrane was tested at permeate fluxes of 20, 40, and 50 LMH, whereas the α-Al_2_O_3_ membrane was tested at permeate fluxes of 20 LMH, 40 LMH, 60 LMH, and 70 LMH.

**Figure 4:**
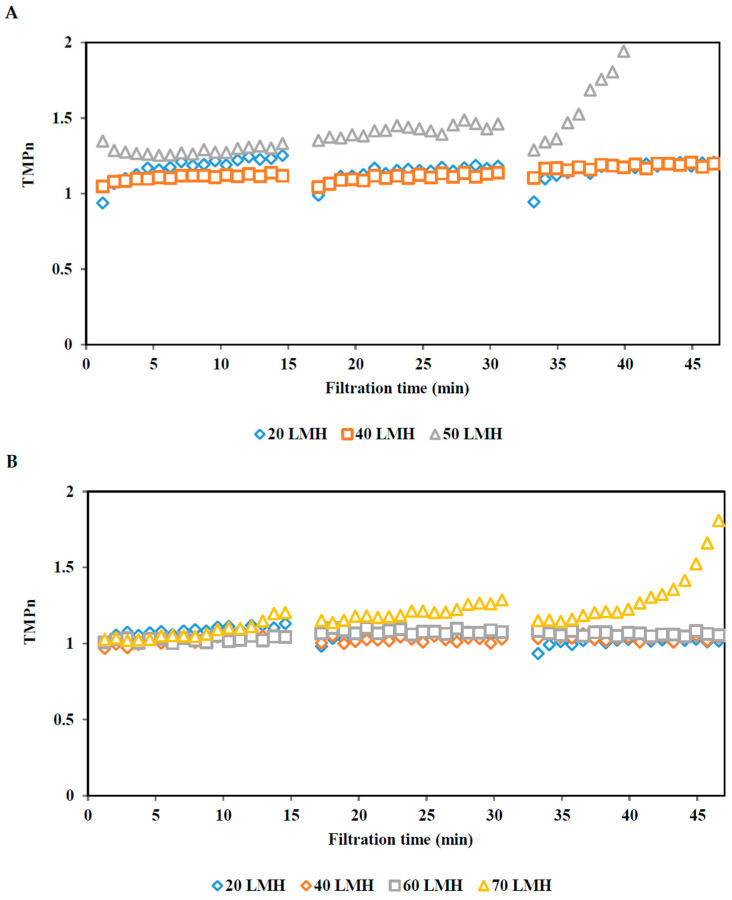
TMP_n_ evolution during constant flux of POME UF at CFV = 0.8 m/s. (**A**) PVDF membrane, and (**B**) α-Al_2_O_3_ membrane.

**Figure 5:**
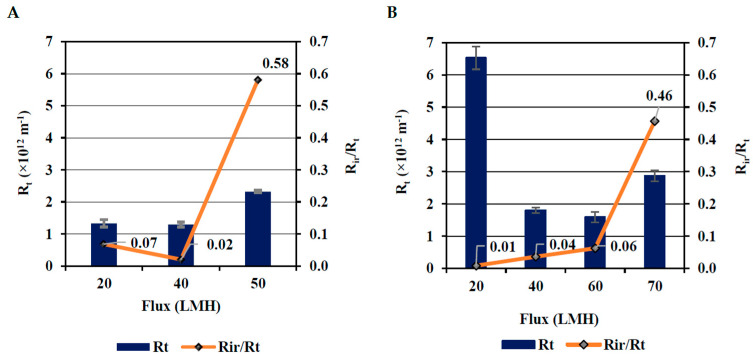
R_t_ and R_ir_/R_t_ variation during constant flux of POME emulsion UF at CFV = 0.8 m/s. (**A**) PVDF membrane, and (**B**) α-Al_2_O_3_ membrane.

Figure 4A shows that the PVDF membrane had a stable TMP_n_ of 1.2 at both 20 LMH and 40 LMH. This observation indicated that either no fouling occurred or there was no filtration resistance from the accumulation of oil droplets. Therefore, backwash was not necessary at 20 LMH and 40 LMH, although it was performed as part of the experimental procedure. However, at 50 LMH, TMP_n_ gradually increased in each filtration cycle, reaching about 1.5 in the second filtration cycle and exponentially increasing in the third filtration cycle, indicating serious fouling. Despite the backwash being performed after the third filtration cycle, it was not effective in stabilizing TMP_n_, suggesting that the fouling was irreversible. This was further supported by the high R_ir_/R_t_ ratio of 0.58 at 50 LMH, compared to 0.07 and 0.02 at 20 LMH and 40 LMH, respectively (Figure 5A).

He, et al. [84] found that for 1500 ppm soybean oil emulsions, the threshold flux for PVDF microfiltration (MF) and PS UF membranes is between 55 and 62 LMH [84]. According to He et al. [84] Stoller et al. [85], and Yang et al. [86], threshold flux is defined as the flux that separates a low fouling region, characterized by a nearly constant rate of fouling (i.e., constant TMP_n_), from a rapid fouling region (i.e., unstable TMP_n_). Therefore, it can be assumed that the threshold flux of PVDF UF membrane treating POME emulsion is approximately 50 LMH. As shown in Figure 4A and Figure 5A, 40 LMH resulted in the least variation in TMP_n_, and correspondingly, the lowest irreversible fouling. Therefore, in this study, 40 LMH was chosen as the optimum condition for the PVDF membrane treating the POME emulsion. It is worth noting that the PVDF membrane showed satisfactory performance at 20 LMH.

On the other hand, the α-Al_2_O_3_ membrane showed stable TMP_n_ values of 1.00 at both 20 and 40 LMH, and 1.08 at 60 LMH, respectively (Figure 4B). This was further supported by the low R_ir_/R_t_ ratio of 0.01, 0.04, and 0.06 at 20 LMH, 40 LMH, and 60 LMH, respectively (Figure 5B). Oil droplets were gradually brought to the membrane surface, but crossflow shear forces enhanced the back diffusion of solutes and prevented buildup, reducing concentration polarization and minimizing the oil concentration on the membrane surface [37,54]. However, at 70 LMH, there was a gradual increase in TMP_n_ in the first and second filtration cycles, reaching 1.3, and an exponential increase in TMP_n_ in the third filtration cycle. This sudden rise in TMP_n_ and the ineffective backwash to stabilize TMP_n_ indicated that serious and rapid fouling occurred due to thickening and compression of the oil layer on the membrane surface wall and a potential distortion of the deposited oil particles [69,79,80]. Oil droplets were brought to the membrane wall surface, forming a layer at a faster rate than they could be removed by the crossflow shear forces [54]. Similar TMP_n_ evolution profiles have been reported in the literature [54,87]. The rapid increase in TMP_n_ likely corresponded to the growth of cake formation [54,88], which increased the resistance of permeate water transport through the membrane pores [54,88]. Further details of the proposed filtration mechanism are available in the Supplementary Materials (Section S4, Figure S1, Table S4). It is also observed in Figure 5B that R_ir_/R_t_ drastically increased to 0.46 at 70 LMH. Therefore, it can be claimed that the threshold flux of the α-Al_2_O_3_ membrane treating POME emulsion is approximately 70 LMH. Due to the low and stable TMP_n_, high fouling resistance, and low R_ir_/R_t_ ratio, the optimum condition of the α-Al_2_O_3_ membrane was selected at 60 LMH. However, satisfactory performance was also observed at 20 and 40 LMH.

It is worth noting that the α-Al_2_O_3_ membrane generally had lower and more stable TMP_n_ than the PVDF membrane at the various permeate fluxes applied. To illustrate, at the optimum condition of 40 LMH applied using PVDF membrane, TMP_n_ reached around 1.2, compared to 1.05 using α-Al_2_O_3_ membrane under at a similar conditions. Furthermore, the optimum condition for the α-Al_2_O_3_ membrane was at 60 LMH, compared to 40 LMH for the PVDF membrane. This difference may be attributed to the surface properties, such as pore size, hydrophilicity, and surface charge, of PVDF and α-Al_2_O_3_ membranes [89]. It was also reported that α-Al_2_O_3_ membranes are more hydrophilic than PVDF membranes [89], indicating that α-Al_2_O_3_ membranes are less prone to fouling. These findings align with the reported literature [5,89]. These observations demonstrate the potential of each membrane and some of the key advantages of using the α-Al_2_O_3_ membrane in the treatment of the POME emulsion.

#### 3.3.2. Effect of Permeate Flux on Fouling Resistances (R)

The fouling behavior of PVDF and α-Al_2_O_3_ membranes was evaluated based on the total filtration resistance (R_t_) and the ratio of irreversible to total resistances (R_ir_/R_t_). R_ir_ and R_t_ were calculated using the measured TMP of each filtration condition. Figure 5 illustrates the results in terms of R_t_ and R_ir_/R_t_. In addition, the filtration resistances (R_m_, R_ir_, and R_r_) for each UF condition were also calculated and reported in the Supplementary Material (Tables S2 and S3).

Generally, increasing the permeate flux to values below the threshold flux resulted in a decrease in the total resistance (R_t_) the over filtration time [69]. To illustrate, for the PVDF membrane, at 20 LMH, and 40 LMH, R_t_ reached 1.34 × 10^12^, and 1.30 × 10^12^ m^−1^, whereas the R_ir_/R_t_ ratio was 0.07 and 0.02, respectively. However, at 50 LMH, the R_t_ and R_ir_/R_t_ ratio increased to 2.32 × 10^12^ m^−1^ and 0.58, respectively, due to the exponential increase in TMP_n_. The increment of filtration resistances can be attributed to pore blocking, causing a severe loss of hydraulic permeability [69].

For the α-Al_2_O_3_ membrane, R_t_ of 6.53 × 10^12^ m^−1^ was observed at 20 LMH, which is higher than those of higher fluxes. This phenomenon could be attributed to the reported trend that increasing the permeate flux below the threshold flux results in decreasing the total resistance (R_t_) over the filtration time [69]. Interestingly, increasing the permeate flux between 40 LMH and 60 LMH resulted in a gradual decrease in R_t_ from 1.80 × 10^12^ to 1.59 × 10^12^ m^−1^, respectively. On the contrary, the R_ir_/R_t_ ratio slightly increased with the permeate flux from 0.01 to 0.06 for permeate fluxes of 20–60 LMH, respectively. However, at 70 LMH, R_t_ increased to 2.86 × 10^12^ m^−1^, with a rise in the R_ir_/R_t_ ratio reaching 0.46. The sudden increase in the R_ir_/R_t_ ratio was supported by the exponential increase in TMP_n_ which indicated serious fouling and a potential pore-blocking [69,90].

The rapid increase in resistance at 50 LMH for PVDF and 70 LMH for α-Al_2_O_3_ membrane could be due to the formation of an oil layer on the membrane surface [54]. During constant flux filtration, fouling appears to be a self-accelerating phenomenon [54]. Miller et al. [54] and Ognier et al. [91] reported that as the membrane pores gradually become blocked by the oil droplets, the local permeate flux in the surrounding pores must compensate to maintain a similar flux over the membrane filtration area. This increase in local permeate flux directs the oil droplets more rapidly into the other open pores, resulting in faster fouling. As more pores are blocked with oil droplets and the local permeate flux is high, cake formation occurs, causing an increase in filtration resistance.

One of the reasons that the optimum permeate flux of the PVDF membrane (40 LMH) is lower than that of the α-Al_2_O_3_ membrane (60 LMH) is the pore size. The PVDF membrane has a pore size of 30 nm, which allows it to capture more oil particles, leading to the accumulation of oil particles on the membrane surface and a thicker oil layer. This phenomenon could cause more severe concentration polarisation on the membrane surface [37,69].

Since the selected optimum conditions for both membranes were below their selected threshold flux, the accumulation of oil layers may not contribute to the membrane resistance [54]. Similar findings were observed by Miller et al. (2014), where increasing the permeate flux did not affect the resistance of the membranes during constant flux filtration below the threshold flux of emulsified oil wastewater and whey protein using PVDF and polysulfone (PS) UF membranes [54].

### 3.4. Efficiency of Membrane Cleaning

PVDF and α-Al_2_O_3_ membranes were cleaned after each UF test using the cleaning methods described in Section 2.4. To recover the hydraulic permeability of the α-Al_2_O_3_ membrane, a stronger chemical cleaning agent (0.75 M NaOH solution) along with heating at 40 °C was required, as the cleaning method used for the PVDF membrane (0.1 M NaOH solution) was not sufficient. The reason for this could be due to the higher likelihood of oil particles being distorted into the larger pores (0.07 µm) of the α-Al_2_O_3_ membrane [69,79,80], compared to the pores of the PVDF membrane (0.03 µm). This can be further supported by the measured PSD of the POME emulsion, which indicated that around 38.5% *v*/*v* have a PSD of 0.04 µm, which is smaller than the pores of the α-Al_2_O_3_ membrane.

**Figure 6:**
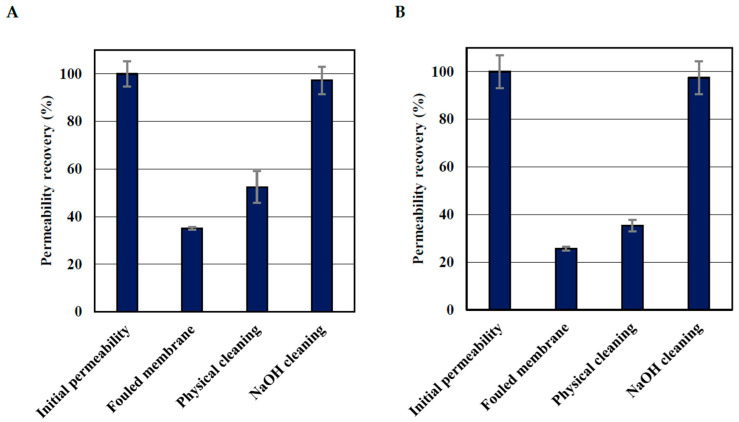
Efficiency of membrane cleaning methods on the recovery of hydraulic permeability (L_h_); (**A**) PVDF membrane and (**B**) α-Al_2_O_3_ membrane.

Figure 6 demonstrates the effectiveness of each cleaning method in recovering the hydraulic permeability of PVDF and α-Al_2_O_3_ membranes. A comparison between the initial and fouled membrane hydraulic permeability conditions was also conducted. Physical cleaning through backwashing and flushing the membranes with demineralized water was able to recover 17.4% and 9.8% of the hydraulic permeability of the PVDF and α-Al_2_O_3_ membranes, respectively. The low efficiency of physical cleaning can be attributed to the hydrophobic nature of the POME emulsion feed, which mainly consists of oil particles that water cannot efficiently remove. Although physical cleaning was not sufficient to fully recover the hydraulic permeability, it indicated that the fouling layer was looser in the PVDF membrane than in the α-Al_2_O_3_ membrane [1]. Chemical cleaning recovered the hydraulic permeability of both membranes, reaching over 97%, confirming that fouling was mostly organic [92]. Additionally, a hydraulic permeability recovery of over 95% is considered indicative of a clean membrane [1].

### 3.5. Comparison Summary of PVDF and α-Al_2_O_3_ Membranes Performance

Table 5 highlights the key findings of the performance comparison between PVDF and α-Al_2_O_3_ membranes in recovering oil and water from the POME emulsion.

**Table 5:** Summary of the performance comparison between PVDF and α-Al_2_O_3_ membranes to treat POME emulsion at 40 LMH and 60 LMH, respectively.

Performance Variables	Experimental Results		Key Findings
	PVDF Membrane	α-Al_2_O_3_ Membrane	
Normalized transmembrane pressure (TMP_n_)	1.2	1.08	α-Al_2_O_3_ membrane showed more stability and slightly lower in magnitude of TMP_n_ at a higher flux than PVDF membrane. At similar fluxes, α-Al_2_O_3_ membrane had TMP_n_ that is lower and more stable than those of PVDF membrane.
Total resistance (R_t_)	1.3 × 10^12^ m^−1^	1.59 × 10^12^ m^−1^	Both membranes showed relatively low total resistance at their respective optimum conditions.
Irreversible to total resistances ratio (R_ir_/R_t_)	0.02	0.06	Both membranes demonstrated minimum irreversible fouling at their respective optimum conditions.
COD rejection (R_j_)	99.8%	99.8%	Both membranes illustrated efficient removal of COD at their respective optimum conditions.
Oil concentration factor (F_o_)	186.8%	253.0%	α-Al_2_O_3_ membrane achieved higher concentration of oil per filtration cycle than PVDF membrane.
Water recovery (R_w_)	46.6%	60.5%	α-Al_2_O_3_ membrane recovered more water per filtration cycle than PVDF membrane.
Membrane cleaning efficiency	97.3%	97.4%	Cleaning methods used in this study achieved an efficient recovery of hydraulic permeability.

## 4. Conclusions

This study provides a detailed comparative assessment of PVDF and α-Al_2_O_3_ ultrafiltration (UF) membranes for the treatment of palm oil mill effluent (POME) emulsion, with a focus on recovering both oil and water. The synthesis of the POME emulsion successfully replicated the characteristics of raw POME, including a fat, oil, and grease (FOG) content of 5.5 ± 0.1 g/L and a particle size distribution (PSD) of 1.54 µm. The optimum UF conditions were determined at permeate fluxes of 40 LMH and 60 LMH using PVDF and α-Al_2_O_3_ membrane, respectively.

Under these conditions, the total resistance (R_t_) was found to be 1.30 × 10^12^ and 1.59 × 10^12^ m^−1^ for PVDF and α-Al_2_O_3_ membranes, respectively. Moreover, the irreversible to total resistances (R_ir_/R_t_) ratio was calculated to be 0.02 and 0.06 for PVDF and α-Al_2_O_3_ membranes, respectively, indicating that most of the developed resistances were reversible and that no severe fouling occurred.

Both membranes demonstrated an effective rejection capacity of COD, reaching 99.8% at their respective optimum UF conditions. The FOG content in POME concentrates, reaching 6.3 g/L and 7.5 g/L for PVDF and α-Al_2_O_3_ membranes, respectively, indicated that it could be suitable for traditional phase separation processes. The produced permeate water from both UF membranes achieved high-quality standards that were superior to the global discharge requirements of POME. Therefore, the permeate water can be discharged into the river system.

The α-Al_2_O_3_ membrane showed more advantages than the PVDF membrane in treating the POME emulsion at their respective optimum UF conditions. For instance, the α-Al_2_O_3_ membrane achieved an oil concentration factor (F_o_) of 253% and a water recovery (R_w_) of 60.5% at 60 LMH, whereas the PVDF membrane achieved F_o_ of 186.8% and R_w_ of 46.6% at 40 LMH. Moreover, α-Al_2_O_3_ membranes are more hydrophilic than PVDF membranes, indicating that α-Al_2_O_3_ membranes are less prone to fouling. Therefore, α-Al_2_O_3_ membranes exhibited lower irreversible fouling at higher flux. For these reasons, α-Al_2_O_3_ membranes are more promising regarding oil and water recovery from POME emulsion, with a more stable performance and a higher rejection capacity.

Hydraulic permeability was efficiently recovered by more than 97% by combining the physical and chemical cleaning of PVDF and α-Al_2_O_3_ membranes at 40 LMH and 60 LMH, respectively. These findings indicate that UF is a viable option for the treatment of POME emulsion, allowing for the recovery of clean water and retention of oil. The UF process for POME could represent a promising method for more sustainable wastewater management in the palm oil industry.

## Figures and Tables

**Table 1:** The properties of PVDF and α-Al_2_O_3_ membranes.

	PVDF Membrane	α-Al_2_O_3_ Membrane
Inner diameter (mm)	5.2	7.0
Pore size (nm) ^a^	30	70
Length (mm)	640.0	600.0
Cross-sectional area (m^2^)	21.2 × 10^−6^	38.5 × 10^−6^
Membrane surface area (m^2^)	1.05 × 10^−2^	1.3 × 10^−2^
Maximum TMP ^b^ (kPa)	500.0	800.0
MWCO ^c^ (kDa)	300.0	500.0
Operating pH range	2–10	2–12
Iso Electric Point	3–4 [62]	8.6–9.8 [63]
Initial Permeability ^a,d^ (LMH·bar)	≥750	≥800 ^e^

^a^ Manufacturer’s design parameters, ^b^ trans-membrane pressure, ^c^ molecular weight cut-off, ^d^ clean water at 25 °C, ^e^ cited from literature [64].

## Data Availability

All relevant data have been reported in the manuscript and the Supplementary Materials. Raw data are not published, but they are available upon request.

## Supplementary Materials

The following supporting information can be downloaded at: https://www.mdpi.com/article/10.3390/membranes15060176/s1, Figure S1: Proposed POME emulsion UF fouling mechanism. (A) Forces on oil droplets near membrane surface, (B) Surface interactions with PVDF membrane, (C) Surface interactions with α-Al_2_O_3_ membrane. F_x_: shear force, F_y1_: surface interactions, and F_y2_: drag force. Table S1: COD calculations of the dominant LCFAs in POME emulsion. Table S2: Filtration resistances variation during constant flux of POME emulsion UF at CFV = 0.8 m/s using PVDF membrane. Table S3: Filtration resistances variation during constant flux of POME emulsion UF at CFV = 0.8 m/s using α-Al_2_O_3_ membrane. Table S4. Summary of the proposed filtration mechanism of PVDF and α-Al_2_O_3_ membranes.

## References

[1] Cifuentes-Cabezas M., Carbonell-Alcaina C., Vincent-Vela M.C., Mendoza-Roca J.A., Álvarez-Blanco S. (2021). Comparison of different ultrafiltration membranes as first step for the recovery of phenolic compounds from olive-oil washing wastewater. Process Saf. Environ. Prot..

[2] Al-Muraisy S.A., Soares L.A., Chuayboon S., Ismail S.B., Abanades S., van Lier J.B., Lindeboom R.E. (2022). Solar-driven steam gasification of oil palm empty fruit bunch to produce syngas: Parametric optimization via central composite design. Fuel Process. Technol..

[3] Avornyo A., Chrysikopoulos C.V. (2024). Applications of graphene oxide (GO) in oily wastewater treatment: Recent developments, challenges, and opportunities. J. Environ. Manag..

[4] Ihsanullah I., Bilal M., Sajid M., Mohammad A.W., Atieh M.A., Ghaffour N. (2024). Emerging MXenes: Revolutionizing oily wastewater treatment—A comprehensive and critical review. Sep. Purif. Technol..

[5] Murić A., Petrinić I., Christensen M.L. (2014). Comparison of ceramic and polymeric ultrafiltration membranes for treating wastewater from metalworking industry. Chem. Eng. J..

[6] Ahmad T., Guria C., Mandal A. (2020). A review of oily wastewater treatment using ultrafiltration membrane: A parametric study to enhance the membrane performance. J. Water Process Eng..

[7] Romero A.S., Innocentini M.D., Oliveira J.V., Lider A., Fey T., Travitzky N., Hotza D. (2024). Unveiling the potential of silicon carbide as a support material and membranes for oily wastewater remediation. Sep. Purif. Technol..

[8] Tian Q., Jiang Y., Li Z., Zhao B., Qiu F., Zhang T. (2024). A dual wastes-based aerogel with inverse beetles-like structure for enhanced oily wastewater treatment. Sep. Purif. Technol..

[9] Sidabutar R., Trisakti B., Michael M., Vanness V., Alexander V., Natasya Y., Alamsyah V., Zaiyat M.Z.Z., Syafriandy S., Al Fath M.T. (2025). Synergistic integration of zeolite engineering and fixed-bed column design for enhanced biogas upgrading: Adsorbent synthesis, CO_2_/CH_4_ separation kinetics, and regeneration assessment. Sep. Purif. Technol..

[10] Saad M.S., Wirzal M.D.H., Putra Z.A. (2021). Review on current approach for treatment of palm oil mill effluent: Integrated system. J. Environ. Manag..

[11] Semilin V., Janaun J., Chung C.H., Touhami D., Haywood S.K., Chong K.P., Yaser A.Z., Zein S.H. (2021). Recovery of oil from palm oil mill effluent using polypropylene micro/nanofiber. J. Hazard. Mater..

[12] Hassan M.A., Farid M.A.A., Zakaria M.R., Ariffin H., Andou Y., Shirai Y. (2024). Palm oil expansion in Malaysia and its countermeasures through policy window and biorefinery approach. Environ. Sci. Policy.

[13] Hosseini S.E., Bagheri G., Wahid M.A., Saat A. (2015). Clean fuel, clean energy conversion technology: Experimental and numerical investigation of palm oil mill effluent biogas flameless combustion. BioResources.

[14] Mahmod S.S., Takriff M.S., AL-Rajabi M.M., Abdul P.M., Gunny A.A.N., Silvamany H., Jahim J.M. (2023). Water reclamation from palm oil mill effluent (POME): Recent technologies, by-product recovery, and challenges. J. Water Process Eng..

[15] Sadhukhan J., Martinez-Hernandez E., Murphy R.J., Ng D.K., Hassim M.H., Ng K.S., Kin W.Y., Jaye I.F.M., Hang M.Y.L.P., Andiappan V. (2018). Role of bioenergy, biorefinery and bioeconomy in sustainable development: Strategic pathways for Malaysia. Renew. Sustain. Energy Rev..

[16] Soo P.L., Bashir M.J., Wong L.-P. (2022). Recent advancements in the treatment of palm oil mill effluent (POME) using anaerobic biofilm reactors: Challenges and future perspectives. J. Environ. Manag..

[17] Gouveia P., Felgueiras F., Mourão Z., Fernandes E.D.O., Moreira A., Gabriel M.F. (2019). Predicting health risk from exposure to trihalomethanes in an Olympic-size indoor swimming pool among elite swimmers and coaches. J. Toxicol. Environ. Health Part A.

[18] Cheng Y.W., Chong C.C., Lam M.K., Leong W.H., Chuah L.F., Yusup S., Setiabudi H.D., Tang Y., Lim J.W. (2021). Identification of microbial inhibitions and mitigation strategies towards cleaner bioconversions of palm oil mill effluent (POME): A review. J. Clean. Prod..

[19] Devlin M., Brodie J. (2023). Nutrients eutrophication. Marine Pollution–Monitoring, Management and Mitigation.

[20] Yusof M.A.B.M., Chan Y.J., Chong C.H., Chew C.L. (2023). Effects of operational processes and equipment in palm oil mills on characteristics of raw Palm Oil Mill Effluent (POME): A comparative study of four mills. Clean. Waste Syst..

[21] Samavati Z., Goh P.S., Ismail A.F., Lau W.J., Samavati A., Ng B.C., Abdullah M.S. (2024). Advancements in membrane technology for efficient POME treatment: A comprehensive review and future perspectives. J. Environ. Sci..

[22] Iskandar M.J., Baharum A., Anuar F.H., Othaman R. (2018). Palm oil industry in South East Asia and the effluent treatment technology—A review. Environ. Technol. Innov..

[23] Cheng Y.W., Chong C.C., Lam M.K., Ayoub M., Cheng C.K., Lim J.W., Yusup S., Tang Y., Bai J. (2021). Holistic process evaluation of non-conventional palm oil mill effluent (POME) treatment technologies: A conceptual and comparative review. J. Hazard. Mater..

[24] Szabo-Corbacho M.A., Pacheco-Ruiz S., Míguez D., Hooijmans C.M., García H.A., Brdjanovic D., van Lier J.B. (2019). Impact of solids retention time on the biological performance of an AnMBR treating lipid-rich synthetic dairy wastewater. Environ. Technol..

[25] Ohimain E.I., Izah S.C. (2017). A review of biogas production from palm oil mill effluents using different configurations of bioreactors. Renew. Sustain. Energy Rev..

[26] Choong Y.Y., Chou K.W., Norli I. (2018). Strategies for improving biogas production of palm oil mill effluent (POME) anaerobic digestion: A critical review. Renew. Sustain. Energy Rev..

[27] Izah S., Oduah A., Ohimain E. (2014). Effects of temperature and fermentation period on the recovery of second grade palm oil from palm press fiber. Int. J. Eng. Sci. Innov. Technol..

[28] Padaki M., Murali R.S., Abdullah M.S., Misdan N., Moslehyani A., Kassim M., Hilal N., Ismail A. (2015). Membrane technology enhancement in oil–water separation. A review. Desalination.

[29] Saeed M.O., Azizli K.A.M., Isa M.H., Ezechi E.H. (2016). Treatment of POME using Fenton oxidation process: Removal efficiency, optimization, and acidity condition. Desalination Water Treat..

[30] Zahrim A., Dexter Z., Joseph C., Hilal N. (2017). Effective coagulation-flocculation treatment of highly polluted palm oil mill biogas plant wastewater using dual coagulants: Decolourisation, kinetics and phytotoxicity studies. J. Water Process Eng..

[31] Bashir M.J., Han T.M., Wei L.J., Aun N.C., Amr S.S.A. (2016). Polishing of treated palm oil mill effluent (POME) from ponding system by electrocoagulation process. Water Sci. Technol..

[32] Das P.P., Sharma M., Purkait M.K. (2022). Recent progress on electrocoagulation process for wastewater treatment: A review. Sep. Purif. Technol..

[33] Azmi N.S., Yunos K.F.M. (2014). Wastewater treatment of palm oil mill effluent (POME) by ultrafiltration membrane separation technique coupled with adsorption treatment as pre-treatment. Agric. Agric. Sci. Procedia.

[34] Hosseini S.E., Wahid M.A. (2015). Pollutant in palm oil production process. J. Air Waste Manag. Assoc..

[35] Yu L., Han M., He F. (2017). A review of treating oily wastewater. Arab. J. Chem..

[36] Yalcinkaya F., Boyraz E., Maryska J., Kucerova K. (2020). A review on membrane technology and chemical surface modification for the oily wastewater treatment. Materials.

[37] Amaral M.C.S., Lebron Y., Moreira V. (2023). Oily wastewater treatment by membrane-assisted technologies. Advanced Technologies in Wastewater Treatment.

[38] Salahi A., Abbasi M., Mohammadi T. (2010). Permeate flux decline during UF of oily wastewater: Experimental and modeling. Desalination.

[39] Asatekin A., Mayes A.M. (2009). Oil industry wastewater treatment with fouling resistant membranes containing amphiphilic comb copolymers. Environ. Sci. Technol..

[40] Zhu X., Tu W., Wee K.-H., Bai R. (2014). Effective and low fouling oil/water separation by a novel hollow fiber membrane with both hydrophilic and oleophobic surface properties. J. Membr. Sci..

[41] Ho Q.N., Lau W.J., Jaafar J., Othman M.H.D., Yoshida N. (2025). Membrane Technology for Valuable Resource Recovery from Palm Oil Mill Effluent (POME): A Review. Membranes.

[42] Aryanti P.T.P., Harsono B., Biantoro M.F.W., Romariyo R., Putri T.A., Hakim A.N., Setia G.A., Saputra D.I., Khoiruddin K. (2025). The role of membrane technology in palm oil mill effluent (POME) decontamination: Current trends and future prospects. J. Environ. Manag..

[43] Alresheedi M.T., Barbeau B., Basu O.D. (2019). Comparisons of NOM fouling and cleaning of ceramic and polymeric membranes during water treatment. Sep. Purif. Technol..

[44] Ilyas A., Vankelecom I.F. (2023). Designing sustainable membrane-based water treatment via fouling control through membrane interface engineering and process developments. Adv. Colloid Interface Sci..

[45] Turk O.K., Zoungrana A., Cakmakci M. (2024). Performances of PTFE and PVDF membranes in achieving the discharge limit of mixed anodic oxidation coating wastewaters treated by membrane distillation. Environ. Sci. Pollut. Res..

[46] Muhamad N.A.S., Mokhtar N.M., Naim R., Lau W.J., Ismail N.H. (2024). Treatment of wastewater from oil palm industry in Malaysia using polyvinylidene fluoride-bentonite hollow fiber membranes via membrane distillation system. Environ. Pollut..

[47] Wadekar S.S., Vidic R.D. (2018). Comparison of ceramic and polymeric nanofiltration membranes for treatment of abandoned coal mine drainage. Desalination.

[48] Lee S.-J., Dilaver M., Park P.-K., Kim J.-H. (2013). Comparative analysis of fouling characteristics of ceramic and polymeric microfiltration membranes using filtration models. J. Membr. Sci..

[49] Subramaniam M., Goh P., Lau W., Tan Y., Ng B., Ismail A. (2017). Hydrophilic hollow fiber PVDF ultrafiltration membrane incorporated with titanate nanotubes for decolourization of aerobically-treated palm oil mill effluent. Chem. Eng. J..

[50] Jiang Y., Xu R., Jiang R., Yang F., Liu H., Sun X. (2025). High-temperature mechanical properties and thermal shock resistance of an alumina-fiber-reinforced alumina ceramic matrix composite. Ceram. Int..

[51] Akash F.A., Shovon S.M., Rahman W., Rahman M.A., Chakraborty P., Prasetya T.A.E., Monir M.U. (2024). Advancements in ceramic membrane technology for water and wastewater treatment: A comprehensive exploration of current utilizations and prospective horizons. Desalination Water Treat..

[52] Sutrisna P.D., Khoiruddin K., Mustika P.C.W., Ismadji S., Wenten I.G. (2024). Advancements in ceramic membranes for robust oil-water separation. J. Environ. Chem. Eng..

[53] Mountoumnjou O., Szymczyk A., Mbambyah E.E.L., Njoya D., Elimbi A. (2022). New low-cost ceramic microfiltration membranes for bacteria removal. Membranes.

[54] Miller D.J., Kasemset S., Paul D.R., Freeman B.D. (2014). Comparison of membrane fouling at constant flux and constant transmembrane pressure conditions. J. Membr. Sci..

[55] Lv M., Feng H., Ding Y., Pan S., Qiao H. (2022). Comparison of the formation, filtration performance, and structural characteristic of self-forming dynamic membranes under constant transmembrane pressure and constant filtration flux. J. Environ. Chem. Eng..

[56] Goh P., Ismail A. (2018). A review on inorganic membranes for desalination and wastewater treatment. Desalination.

[57] Khadaroo S.N., Grassia P., Gouwanda D., Poh P.E. (2019). Is the dewatering of Palm Oil Mill Effluent (POME) feasible? Effect of temperature on POME’s rheological properties and compressive behavior. Chem. Eng. Sci..

[58] Zhong Z., Xing W., Zhang B. (2013). Fabrication of ceramic membranes with controllable surface roughness and their applications in oil/water separation. Ceram. Int..

[59] Xu Y., Li Y., Hou Y. (2019). Reducing ultrafiltration membrane fouling during recycled paper mill wastewater treatment using pretreatment technologies: A comparison between coagulation and Fenton. J. Chem. Technol. Biotechnol..

[60] Moser P.B., Ricci B.C., Reis B.G., Neta L.S., Cerqueira A.C., Amaral M.C. (2018). Effect of MBR-H_2_O_2_/UV Hybrid pre-treatment on nanofiltration performance for the treatment of petroleum refinery wastewater. Sep. Purif. Technol..

[61] Elhady S., Bassyouni M., Mansour R.A., Elzahar M.H., Abdel-Hamid S., Elhenawy Y., Saleh M.Y. (2020). Oily wastewater treatment using polyamide thin film composite membrane technology. Membranes.

[62] Nguyen M., Loulergue P., Karpel N., Teychene B. (2019). Electron beam irradiation of polyvinylidene fluoride/polyvinylpyrrolidone ultrafiltration membrane in presence of zwitterions molecules evaluation of filtration performances. Radiat. Phys. Chem..

[63] Kobayashi Y., Yasuda Y., Morita T. (2020). Low-temperature synthesis of α-alumina based on sol-gel processes. Adv. Mater. Process Technol..

[64] Liu Y., Zhu W., Guan K., Peng C., Wu J. (2018). Preparation of high permeable alumina ceramic membrane with good separation performance via UV curing technique. RSC Adv..

[65] Chen M., Shang R., Sberna P.M., Luiten-Olieman M.W., Rietveld L.C., Heijman S.G. (2020). Highly permeable silicon carbide-alumina ultrafiltration membranes for oil-in-water filtration produced with low-pressure chemical vapor deposition. Sep. Purif. Technol..

[66] Chen M., Heijman S.G., Luiten-Olieman M.W., Rietveld L.C. (2022). Oil-in-water emulsion separation: Fouling of alumina membranes with and without a silicon carbide deposition in constant flux filtration mode. Water Res..

[67] Xing J., Liang H., Chuah C.J., Bao Y., Luo X., Wang T., Wang J., Li G., Snyder S.A. (2019). Insight into Fe (II)/UV/chlorine pretreatment for reducing ultrafiltration (UF) membrane fouling: Effects of different natural organic fractions and comparison with coagulation. Water Res..

[68] Elmaleh S., Abdelmoumni L. (1997). Cross-flow filtration of an anaerobic methanogenic suspension. J. Membr. Sci..

[69] Ren Q., Chen X., Yumminaga Y., Wang N., Yan W., Li Y., Liu L., Shi J. (2021). Effect of operating conditions on the performance of multichannel ceramic ultrafiltration membranes for cattle wastewater treatment. J. Water Process Eng..

[70] Hernández K., Muro C., Ortega R.E., Velazquez S., Riera F. (2021). Water recovery by treatment of food industry wastewater using membrane processes. Environ. Technol..

[71] APHA (2005). Standard Methods for the Examination of Water and Wastewater. APHA, American Water Works Association and Water Environment Federation.

[72] Rupani P.F., Singh R.P., Ibrahim M.H., Esa N. (2010). Review of current palm oil mill effluent (POME) treatment methods: Vermicomposting as a sustainable practice. World Appl. Sci. J..

[73] Ahmad A.L., Ismail S., Bhatia S. (2003). Water recycling from palm oil mill effluent (POME) using membrane technology. Desalination.

[74] Okogbenin O., Anisiobi G., Okogbenin E., Okunwaye T., Ojieabu A. (2014). Microbiological assessment and physiochemical parameters of palm oil mill effluent collected in a local mill in Ovia North East area of Edo State, Nigeria. Her. J. Microbiol. Biotechnol..

[75] Kanani D.M., Sun X., Ghosh R. (2008). Reversible and irreversible membrane fouling during in-line microfiltration of concentrated protein solutions. J. Membr. Sci..

[76] Bennani C.F., Ousji B., Ennigrou D.J. (2015). Reclamation of dairy wastewater using ultrafiltration process. Desalination Water Treat..

[77] Bortoluzzi A.C., Faitão J.A., Di Luccio M., Dallago R.M., Steffens J., Zabot G.L., Tres M.V. (2017). Dairy wastewater treatment using integrated membrane systems. J. Environ. Chem. Eng..

[78] Huang B., Gu H., Xiao K., Qu F., Yu H., Wei C. (2020). Fouling mechanisms analysis via combined fouling models for surface water ultrafiltration process. Membranes.

[79] Tonova K., Lazarova M., Dencheva-Zarkova M., Paniovska S., Tsibranska I., Stanoev V., Dzhonova D., Genova J. (2020). Separation of glucose, other reducing sugars and phenolics from natural extract by nanofiltration: Effect of pressure and cross-flow velocity. Chem. Eng. Res. Des..

[80] Cifuentes-Cabezas M., Vincent-Vela M.C., Mendoza-Roca J.A., Álvarez-Blanco S. (2022). Use of ultrafiltration ceramic membranes as a first step treatment for olive oil washing wastewater. Food Bioprod. Process..

[81] Guo C., Chang H., Liu B., He Q., Xiong B., Kumar M., Zydney A.L. (2018). A combined ultrafiltration–reverse osmosis process for external reuse of Weiyuan shale gas flowback and produced water. Environ. Sci. Water Res. Technol..

[82] Kamyab H., Chelliapan S., Din M.F.M., Rezania S., Khademi T., Kumar A. (2018). Palm oil mill effluent as an environmental pollutant. Palm Oil.

[83] Azmi N.S., Yunos K.F.M., Baharuddin A.S., Dom Z.M. (2013). The effect of operating parameters on ultrafiltration and reverse osmosis of palm oil mill effluent for reclamation and reuse of water. BioResources.

[84] He Z., Miller D.J., Kasemset S., Paul D.R., Freeman B.D. (2017). The effect of permeate flux on membrane fouling during microfiltration of oily water. J. Membr. Sci..

[85] Stoller M., Ochando-Pulido J.M. (2014). About merging threshold and critical flux concepts into a single one: The boundary flux. Sci. World J..

[86] Yang Q., Luo J., Guo S., Hang X., Chen X., Wan Y. (2019). Threshold flux in concentration mode: Fouling control during clarification of molasses by ultrafiltration. J. Membr. Sci..

[87] Cai Z., Wee C., Benjamin M.M. (2013). Fouling mechanisms in low-pressure membrane filtration in the presence of an adsorbent cake layer. J. Membr. Sci..

[88] Cifuentes-Cabezas M., Bohórquez-Zurita J.L., Gil-Herrero S., Vincent-Vela M.C., Mendoza-Roca J.A., Álvarez-Blanco S. (2023). Deep study on fouling modelling of ultrafiltration membranes used for OMW treatment: Comparison between semi-empirical models, response surface, and artificial neural networks. Food Bioprocess Technol..

[89] Jeong Y., Kim Y., Jin Y., Hong S., Park C. (2018). Comparison of filtration and treatment performance between polymeric and ceramic membranes in anaerobic membrane bioreactor treatment of domestic wastewater. Sep. Purif. Technol..

[90] Lv J., Zhang G., Zhang H., Zhao C., Yang F. (2018). Improvement of antifouling performances for modified PVDF ultrafiltration membrane with hydrophilic cellulose nanocrystal. Appl. Surf. Sci..

[91] Ognier S., Wisniewski C., Grasmick A. (2004). Membrane bioreactor fouling in sub-critical filtration conditions: A local critical flux concept. J. Membr. Sci..

[92] Dereli R.K., Heffernan B., Grelot A., van der Zee F.P., van Lier J.B. (2015). Influence of high lipid containing wastewater on filtration performance and fouling in AnMBRs operated at different solids retention times. Sep. Purif. Technol..

