# Supplementary material for: Oil and Water Recovery from Palm Oil Mill Effluent: A Comparative Study of PVDF and α-Al2O3 Ultrafiltration Membranes
Source: Membranes (Basel). 2025 Jun 10;15(6):176. doi: 10.3390/membranes15060176 (PMC12194983; doi:10.3390/membranes15060176)
Supplement: Supplementary file 1 [file membranes-15-00176-s001.zip › membranes-3638960-supplementary.pdf]

Supplementary Material

Oil and water recovery from palm oil mill effluent: A comparative study of PVDF and α-Al_2O_3 ultrafiltration membranes

Saqr A.A. Al-Muraisy, Jiamin Wu, Mingliang Chen, Begüm Tanis, Sebastiaan G.J. Heijman, Shahrul bin Ismail, Jules B. van Lier and Ralph E.F. Lindeboom

1. Phases of the UF filtration cycle

Each filtration cycle consisted of 5 phases:

1. Permeability test with demineralised water at a constant flux for 15 minutes. This step takes place before the first cycle of POME emulsion filtration.
2. Forward flush with POME emulsion feed for 15 seconds at a cross-flow velocity of 0.8 m/s to fill the filtration loop and remove air bubbles.
3. Ultrafiltration of POME emulsion at a constant flux for 15 minutes.
4. Backwash the membrane module with demineralised water at a fixed pressure of 3 bar for 30 seconds to remove the hydraulically reversible fouling.
5. Permeability test with demineralised water at a constant flux for 15 minutes. This step takes place after the last cycle of POME emulsion filtration.

Each permeability test cycle consisted of 2 phases:

1. Forward flush with demineralised water for 15 seconds at a cross flow velocity of 0.8 m/s to fill the filtration loop and remove air bubbles.
2. Filtration of demineralised water at a constant flux for 15 minutes.

2. Ratio of COD to FOG concentration

To determine the FOG concentration in POME based on the COD value, the theoretical ratio of g COD/ g FOG is calculated. Since lipids in POME emulsion are primarily fats, they are considered triglycerides. This means that 1 mole of FOG is composed of 1 mole of glycerol and 3 moles of Long Chain Fatty Acids (LCFA). The volumetric compositions of the five quantitatively dominant LCFAs in POME emulsion were selected as representative [1] as shown in Table S.1.

The average molecular weight (MW) of the LCFAs was calculated using equation S.1, considering the MW and volumetric composition of each LCFA. The ratio of COD to the average mass of LCFA was then calculated using equation S.2. Finally, the overall ratio of COD to FOG mass was determined to be 2.71 g COD/g FOG, as calculated using equation S.3.

Table S.1. COD calculations of the dominant LCFAs in POME emulsion.

Parameter	Unit	Glycerol	Palmitic acid	Oleic acid	Myristic acid	Stearic acid	Linoleic acid
Composition [2]	% v/v	-	22.45	14.54	12.66	10.41	9.53
Molecular weight (MW)	g/mol	92	256	282	228	284	280
Theoretical chemical oxygen demand (COD)	g COD/mol	112	736	816	640	832	800
COD/mass ratio	g COD/g substance	1.217	2.875	2.894	2.807	2.930	2.857

$$MW_{LCFA\ avg} = \frac{\sum(MW_{LCFA} \times Composition\ (\%)_{LCFA})}{\sum Composition\ (\%)_{LCFA}} = 270.16\ g/mol \quad (S.1)$$

$$\frac{COD}{LCFA\ avg}\ mass\ ratio = \frac{\sum(\frac{COD}{mass} ratio_{LCFA} \times omposition\ (\%)_{LCFA})}{\sum Composition\ (\%)_{LCFA}} = 2.88\ g\ COD/\ g\ LCFA_{avg} \quad (S.2)$$

$$\frac{COD}{FOG}\ mass\ ratio = \frac{1\ mol \times MW_{glc.} \times \frac{COD}{mass_{glc.}} + 3\ mol \times MW_{LCAF\ avg} \times \frac{COD}{mass_{LCFA}}}{1\ mol \times MW_{glc.} + 3\ mol \times MW_{LCAF\ avg}} \quad (S.3)$$

$$= \mathbf{2.71\ g\ COD/\ g\ FOG}$$

3. Fouling resistances (R)

Resistance-in-series model was used to calculate the various types of membrane resistances as illustrated in equations (S.4 – S.7).

$$R_m = \frac{TMP_{n,0}}{\mu J} \quad (S.4)$$

$$R_t = \frac{TMP_{n,1}}{\mu J} \quad (S.5)$$

$$R_{ir} = \frac{TMP_{n,2}}{\mu J} - \frac{TMP_{n,0}}{\mu J}$$

(S.6)

$$R_r = R_t - R_m - R_{ir} \quad (S.7)$$

where R_t (m^{-1}) is defined as the total resistance comprising intrinsic membrane resistance (R_m, m^{-1}), hydraulic reversible resistance (R_r, m^{-1}) and irreversible resistance (R_{ir}, m^{-1}). J is the permeate flux (m/s). μ is the permeate water dynamic viscosity which was assumed to be equal to that of pure water [3] (9.544×10^{-4} Pa· s) at the filtration temperature of 22°C. R_m was identified via the hydraulic permeability test of demineralised water before the first cycle of each UF experiment (Equation S.4). $TMP_{n,o}$ is the demineralised water filtration pressure before UF experiment. R_t was calculated based on the final filtration pressure of POME emulsion ($TMP_{n,1}$) as shown in Equation S.5. After backwash, the average TMP_n of hydraulic permeability test was recorded as $TMP_{n,2}$, and R_{ir} was determined from Equation S.6.

For each UF experiment using PVDF and α-Al_2O_3 membranes, the fouling behavior was evaluated by examining the membrane's intrinsic resistance (R_m), reversible resistance (R_r), and irreversible resistance (R_{ir}), and the total resistance (R_t). Tables S.2 and S.3 display the variation in filtration resistances at different permeate fluxes for PVDF and α-Al_2O_3 membranes.

It can be observed from Table S.2 that R_m is inversely proportional to the permeate flux. When using the PVDF membrane, R_m decreased from $1.11 \pm 0.10 \times 10^{12}$ m^{-1} at a permeate flux of 20 LMH to $0.80 \pm 0.03 \times 10^{12}$ m^{-1} at 50 LMH. On the other hand, R_{ir} increased proportionally with the permeate flux, rising from $(0.09 \pm 0.01) \times 10^{12}$ m^{-1} at 20 LMH to $(1.35 \pm 0.03) \times 10^{12}$ m^{-1} at 50 LMH. However, at 40 LMH, a minimum R_{ir} of $(0.03 \pm 0.03) \times 10^{12}$ m^{-1} was observed. R_r did not show big changes across the range of permeate fluxes,

ranging (0.13-0.18) × 10^{12} m^{-1}. These observations support the selection of 40 LMH as the optimum condition for treating POME emulsion using the PVDF membrane.

Similarly, R_m showed a declining trend with increasing the permeate flux when using α-Al_2O_3 membrane. For instance, R_m decreased from (6.26 ± 0.24) × 10^{12} m^{-1} at a permeate flux of 20 LMH to (1.53 ± 0.14) × 10^{12} m^{-1} at 70 LMH. Furthermore, R_{ir} slightly increased proportionally with permeate flux, rising from (0.05 ± 0.04) × 10^{12} m^{-1} at 20 LMH to (0.10 ± 0.01) × 10^{12} m^{-1} at 60 LMH. However, at 70 LMH, R_{ir} increased drastically to (1.31 ± 0.02) × 10^{12} m^{-1}, indicating severe fouling. Additionally, R_r did not show big changes from 20 to 60 LMH, ranging ranging (0.10 - 0.21) × 10^{12} m^{-1}, but decreased to 0.03 ± 0.01 × 10^{12} m^{-1} at 70 LMH. These findings support the conclusion that serious fouling and potential pore blocking occurred at 70 LMH [4]. Therefore, the selection of 60 LMH as the optimum condition for treating POME emulsion using the α-Al_2O_3 membrane is further validated.

Table S.2: Filtration resistances variation during constant flux of POME emulsion UF at CFV = 0.8 m/s using PVDF membrane.

Permeate flux (LMH)	R_m	R_t	R_{ir}	R_r	R_{ir}/R_t
	(×10^{12} m^{-1})				
20	1.11 ± 0.10	1.34 ± 0.11	0.09 ± 0.01	0.13 ± 0.02	0.07
40	1.09 ± 0.06	1.30 ± 0.08	0.03 ± 0.03	0.18 ± 0.05	0.02
50	0.80 ± 0.03	2.32 ± 0.04	1.35 ± 0.03	0.17 ± 0.04	0.58

Table S.3: Filtration resistances variation during constant flux of POME emulsion UF at CFV = 0.8 m/s using α-Al_2O_3 membrane.

Permeate flux (LMH)	R_m	R_t	R_{ir}	R_r	R_{ir}/R_t
	(×10^{12} m^{-1})				
20	6.26 ± 0.24	6.53 ± 0.35	0.05 ± 0.04	0.21 ± 0.07	0.01
40	1.65 ± 0.01	1.80 ± 0.09	0.07 ± 0.01	0.10 ± 0.07	0.04
60	1.33 ± 0.15	1.59 ± 0.16	0.10 ± 0.01	0.16 ± 0.004	0.06

70	1.53 ± 0.14	2.86 ± 0.17	1.31 ± 0.02	0.03 ± 0.01	0.46

4. Proposed filtration mechansims

The fouling behavior of PVDF and α-Al_2O_3 membranes during ultrafiltration of POME emulsions can be explained by analyzing the dominant interfacial forces and physicochemical interactions between the oil droplets and the membrane surface. Figure S.1(A) schematically illustrates the primary forces acting on oil droplets within the membrane module: the tangential shear force (F_x), surface interaction forces (F_{y1}), and vertical drag force (F_{y2}), as described in previous studies [5-7]. These forces arise from the imposed crossflow velocity (CFV = 0.8 m/s), electrostatic interactions, steric effects, and the convective flow of permeate water. F_x acts along the direction of the feed flow, while F_{y1} and F_{y2} influence the droplet behaviour normal to the membrane surface. The balance between these forces governs droplet deposition, accumulation, and removal during filtration [5]. Figures S.1(B and C) further elucidate the proposed surface interactions with PVDF and α-Al_2O_3 membranes, respectively, as well as the interactions of oil droplets within POME emulsion.

PVDF membrane has an IEP of 3 - 4 [8] (Table 1). Therefore, oil droplets with a pH of 5.4 are electrostatically repelled from the membrane surface due to their similar negative electrostatic charge, as shown in Figure S.1(B). However, as the oil concentration increases during filtration and permeate water removal, electrostatic repulsion is surpassed by F_{y2}, resulting in the formation of an oil layer on the membrane surface, as reported in previous studies [5, 9, 10]. The oil layer develops steric hindrance on the membrane surface, enhancing the rejection capacity of oil and stabilised TMP_n. Steric hindrance is a result of intermolecular reactions of oil droplets and is often used to improve selectivity [11]. At 20 LMH, TMP_n of PVDF membrane was not stable in the first filtration cycle but stabilised in the second and third cycles, which could be attributed to the gradual formation of the oil layer on the membrane

surface. The formation of the oil layer on PVDF membrane surface due to F_{y2} was balanced by the shear-induced removal due to F_x [5]. Furthermore, the balance between F_{y2} and F_x prevented the continuous formation of the oil droplets beyond a critical size that may result in critical fouling and membrane clogging [5]. In this dynamic process, smaller-size coalesce of oil droplets that were not removed by F_x played a role in the size reduction of membrane pores and the increase of COD rejection. Increasing the permeate flux results in increasing F_{y2}, which compresses the accumulated oil layer on the membrane surface, leading to the dominance of irreversible fouling, as illustrated in Figure S.1(B).

α-Al_2O_3 membrane has an IEP of 8.6 - 9.8 [12] (Table 1). Therefore, it is positively charged when treating POME emulsion. Consequently, oil droplets are electrostatically attracted to the membrane surface. The electrostatic attraction allowed the adsorption of oil droplets, leading to the formation of an oil layer on α-Al_2O_3 membrane surface. This phenomenon altered the membrane charge from positive to negative, as illustrated in Figure S.1(C). The theory of charge inversion of membrane surface caused by the fouling layer is supported by various reported studies [13, 14], which have found that foulant layers shift the interaction between oil droplets and the membrane surface to interactions between oil droplets and the oil layer. As a result, the electrostatic forces change from attractive to repulsive, hindering further adsorption of oil droplets. Due to the repulsive surface interactions between membrane surface-oil droplet and deposited oil layer-oil droplet [5], these types of surface interactions contribute to fouling mitigation. The summarised findings of the proposed filtration mechanism are illustrated in Table S.4. To validate these proposed mechanisms, it is recommended to utilise the application of high speed imaging to study the behaviour and dynamics of oil droplets in tubular membranes [15]. However, it is important to note that this validation was not conducted in the present study.

A

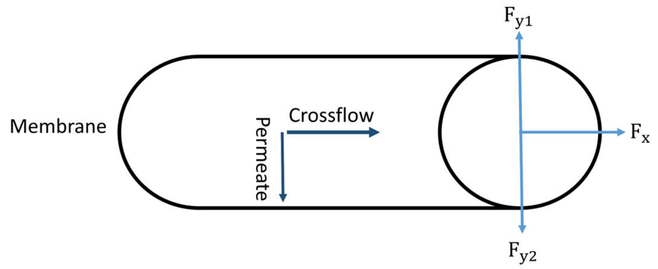

B

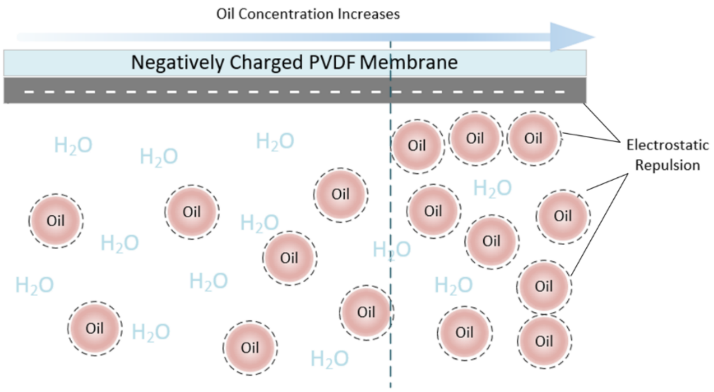

C

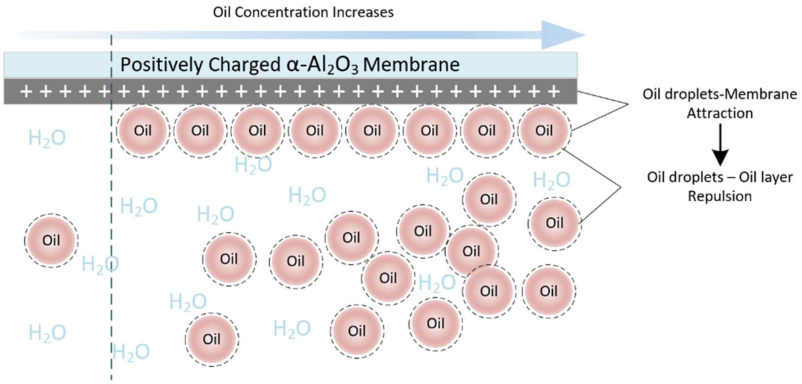

Figure S.1. Proposed POME emulsion UF fouling mechanism. A) Forces on oil droplets near membrane surface, B) Surface interactions with PVDF membrane, C) Surface interactions with α-Al2O3 membrane. F_x: shear force, F_{y1}: surface interactions, and F_{y2}: drag force.

Table S.4. Summary of the proposed filtration mechanism of PVDF and α-Al_2O_3 membranes.

Membrane	Proposed filtration mechanism
PVDF membrane	Initially, oil droplets are electrostatically repelled from the membrane surface. However, as the oil concentration increases, this repulsion is surpassed, leading to the deposition of oil on the membrane surface. The formation of an oil layer on the membrane surface, caused by F_{y2}, is balanced by F_x, which helps mitigate fouling.

α-Al_2O_3 membrane	Oil droplets are electrostatically attracted to the membrane surface, forming an oil layer which caused charge inversion. This phenomenon hindered further adsorption of oil droplets. The formation of the oil layer on the membrane surface, due to F_{y2}, is balanced by F_x, which helps mitigate fouling.

REFERENCES

[1] W. Yoochatchaval, S. Kumakura, D. Tanikawa, T. Yamaguchi, M. Yunus, S. Chen, K. Kubota, H. Harada, K. Syutsubo, Anaerobic degradation of palm oil mill effluent (POME), Water Science and Technology, 64 (2011) 2001-2008.

[2] A. Mancini, E. Imperlini, E. Nigro, C. Montagnese, A. Daniele, S. Orrù, P. Buono, Biological and nutritional properties of palm oil and palmitic acid: effects on health, Molecules, 20 (2015) 17339-17361.

[3] D.J. Miller, S. Kasemset, D.R. Paul, B.D. Freeman, Comparison of membrane fouling at constant flux and constant transmembrane pressure conditions, Journal of Membrane Science, 454 (2014) 505-515.

[4] Q. Ren, X. Chen, Y. Yumminaga, N. Wang, W. Yan, Y. Li, L. Liu, J. Shi, Effect of operating conditions on the performance of multichannel ceramic ultrafiltration membranes for cattle wastewater treatment, Journal of Water Process Engineering, 41 (2021) 102102.

[5] Z. He, S. Kasemset, A.Y. Kirschner, Y.-H. Cheng, D.R. Paul, B.D. Freeman, The effects of salt concentration and foulant surface charge on hydrocarbon fouling of a poly (vinylidene fluoride) microfiltration membrane, Water research, 117 (2017) 230-241.

[6] C.Y. Tang, Y.-N. Kwon, J.O. Leckie, The role of foulant–foulant electrostatic interaction on limiting flux for RO and NF membranes during humic acid fouling—theoretical basis, experimental evidence, and AFM interaction force measurement, Journal of Membrane Science, 326 (2009) 526-532.

[7] S. Vigneswaran, D.-Y. Kwon, Effect of ionic strength and permeate flux on membrane fouling: Analysis of forces acting on particle deposit and cake formation, KSCE Journal of Civil Engineering, 19 (2015) 1604-1611.

[8] M. Nguyen, P. Loulergue, N. Karpel, B. Teychene, Electron beam irradiation of polyvinylidene fluoride/polyvinylpyrrolidone ultrafiltration membrane in presence of zwitterions molecules evaluation of filtration performances, Radiation Physics and Chemistry, 159 (2019) 101-110.

[9] Z. He, D.J. Miller, S. Kasemset, L. Wang, D.R. Paul, B.D. Freeman, Fouling propensity of a poly (vinylidene fluoride) microfiltration membrane to several model oil/water emulsions, Journal of Membrane Science, 514 (2016) 659-670.

[10] E.N. Tummons, V.V. Tarabara, J.W. Chew, A.G. Fane, Behavior of oil droplets at the membrane surface during crossflow microfiltration of oil–water emulsions, Journal of Membrane Science, 500 (2016) 211-224.

[11] P. Sun, R. Ma, H. Deng, Z. Song, Z. Zhen, K. Wang, T. Sasaki, Z. Xu, H. Zhu, Intrinsic high water/ion selectivity of graphene oxide lamellar membranes in concentration gradient-driven diffusion, Chemical science, 7 (2016) 6988-6994.

[12] Y. Kobayashi, Y. Yasuda, T. Morita, Low-temperature synthesis of α-alumina based on sol-gel processes, Advances in Materials and Processing Technologies, (2020) 1-32.

[13] N. Ochoa, M. Masuelli, J. Marchese, Effect of hydrophilicity on fouling of an emulsified oil wastewater with PVDF/PMMA membranes, Journal of Membrane Science, 226 (2003) 203-211.

[14] L. Zou, I. Vidalis, D. Steele, A. Michelmore, S. Low, J. Verberk, Surface hydrophilic modification of RO membranes by plasma polymerization for low organic fouling, Journal of Membrane Science, 369 (2011) 420-428.

[15] R. Lindeboom, G. Smith, D. Jeison, H. Temmink, J.B. van Lier, Application of high speed imaging as a novel tool to study particle dynamics in tubular membrane systems, Journal of Membrane Science, 368 (2011) 95-99.
